# ATP from synaptic terminals and astrocytes regulates NMDA receptors and synaptic plasticity through PSD-95 multi-protein complex

**DOI:** 10.1038/srep33609

**Published:** 2016-09-19

**Authors:** U. Lalo, O. Palygin, A. Verkhratsky, S. G. N. Grant, Y. Pankratov

**Affiliations:** 1 The University of Warwick, School of Life Sciences, Coventry, UK; 2 The University of Manchester, Faculty of Life Science, Manchester, UK; 3 Centre for Clinical Brain Science, Edinburgh University, Edinburgh, UK

## Abstract

Recent studies highlighted the importance of astrocyte-secreted molecules, such as ATP, for the slow modulation of synaptic transmission in central neurones. Biophysical mechanisms underlying the impact of gliotransmitters on the strength of individual synapse remain, however, unclear. Here we show that purinergic P2X receptors can bring significant contribution to the signalling in the individual synaptic boutons. ATP released from astrocytes facilitates a recruitment of P2X receptors into excitatory synapses by Ca^2+^-dependent mechanism. P2X receptors, co-localized with NMDA receptors in the excitatory synapses, can be activated by ATP co-released with glutamate from pre-synaptic terminals and by glia-derived ATP. An activation of P2X receptors in turn leads to down-regulation of postsynaptic NMDA receptors via Ca^2+^-dependent de-phosphorylation and interaction with PSD-95 multi-protein complex. Genetic deletion of the PSD-95 or P2X4 receptors obliterated ATP-mediated down-regulation of NMDA receptors. Impairment of purinergic modulation of NMDA receptors in the PSD-95 mutants dramatically decreased the threshold of LTP induction and increased the net magnitude of LTP. Our findings show that synergistic action of glia- and neurone-derived ATP can pre-modulate efficacy of excitatory synapses and thereby can have an important role in the glia-neuron communications and brain meta-plasticity.

In the CNS, ATP is released from the nerve terminals (mainly by exocytosis) and astrocytes (both by vesicular release and diffusion through plasmalemmal channels)^1^,^2^,^3^. Release of ATP represents a powerful pathway of glia-neuron interaction implicated in the synaptic plasticity^4^,^5^,^6^, metaplasticity^7^ and neurological disorders^2^,^8^. Action of ATP on neurones is mediated by metabotropic P2Y receptors and ionotropic P2X receptors (P2XRs) abundantly expressed in the brain^1^,^9^. Although the role for purinoceptors in synaptic signalling was suggested in the late 70s^1^, ATP-mediated synaptic currents were discovered relatively recently in several brain areas, such as medial habenula^10^, hippocampus^11^ and somatosensory cortex^12^. Physiological importance of the P2X receptors is highlighted by their high calcium permeability^9^,^10^,^11^,^12^ and dynamic interaction with other ligand-gated channels^13^,^14^.

The P2X receptors, activated by ATP released from astrocytes, can modulate excitatory synapses in the hippocampal and magnocellular neurones^15^,^16^ as well as GABAergic inhibitory synapses in the neocortical neurones^3^. In addition to P2XR-mediated modulation of synaptic strength at the postsynaptic level, glia-derived ATP can exert a widespread pre-synaptic action upon diffusion and extracellular breakdown to adenosine and activation of pre-synaptic A1 receptors^1^,^5^. Hence, ATP is a quintessential glio-signalling molecule for slow long-range modulation of synaptic strength^2^,^7^.

The physiological contribution of postsynaptic P2XR-mediated signals to excitatory neurotransmission remains elusive^8^, as widespread expression of P2X receptors in the brain^1^,^8^,^9^ contrasts with their generally small contribution to the net excitatory input^11^,^12^. Moreover, there is also a disparity between capability of P2X receptors to mediate fast excitatory synaptic input^9^,^10^,^11^,^12^,^17^ and the slow time-scale of purinergic modulation of excitatory synaptic transmission^15^,^16^. Identification of P2XR-mediated modulation of the excitatory synapses^15^,^16^ was not supported by the direct evidence for P2XR-mediated signals. It is, therefore, unclear how can extracellular ATP, spread by diffusion, produce a *localised* effect on the *individual* excitatory synapse. Finally, the influence of P2XRs on crucial element of synaptic plasticity – the NMDA receptors – is yet to be studied.

In the on-going debate on physiological significance of gliotransmission, ATP is often considered merely as a precursor of adenosine, which modulates synaptic transmission and plasticity via pre-synaptic mechanisms^2^,^5^,^7^. In the present paper, we endeavoured to bridge the glia-neuron interaction and purinergic regulation of synaptic strength. We used several experimental approaches to dissect specific roles for synaptic and glial release of ATP. We show an importance of cooperation between synaptic and glial exocytosis of ATP for the activation of fast purinergic signalling at the individual cortical synapse. We demonstrate that P2X receptors regulate of NMDA receptors through the PSD-95 multi-protein complex and show the importance of this pathway for the synaptic meta-plasticity.

## Results

### Release of ATP from synaptic terminals and localisation of P2X receptors in the excitatory synapses

**Figure 1:**
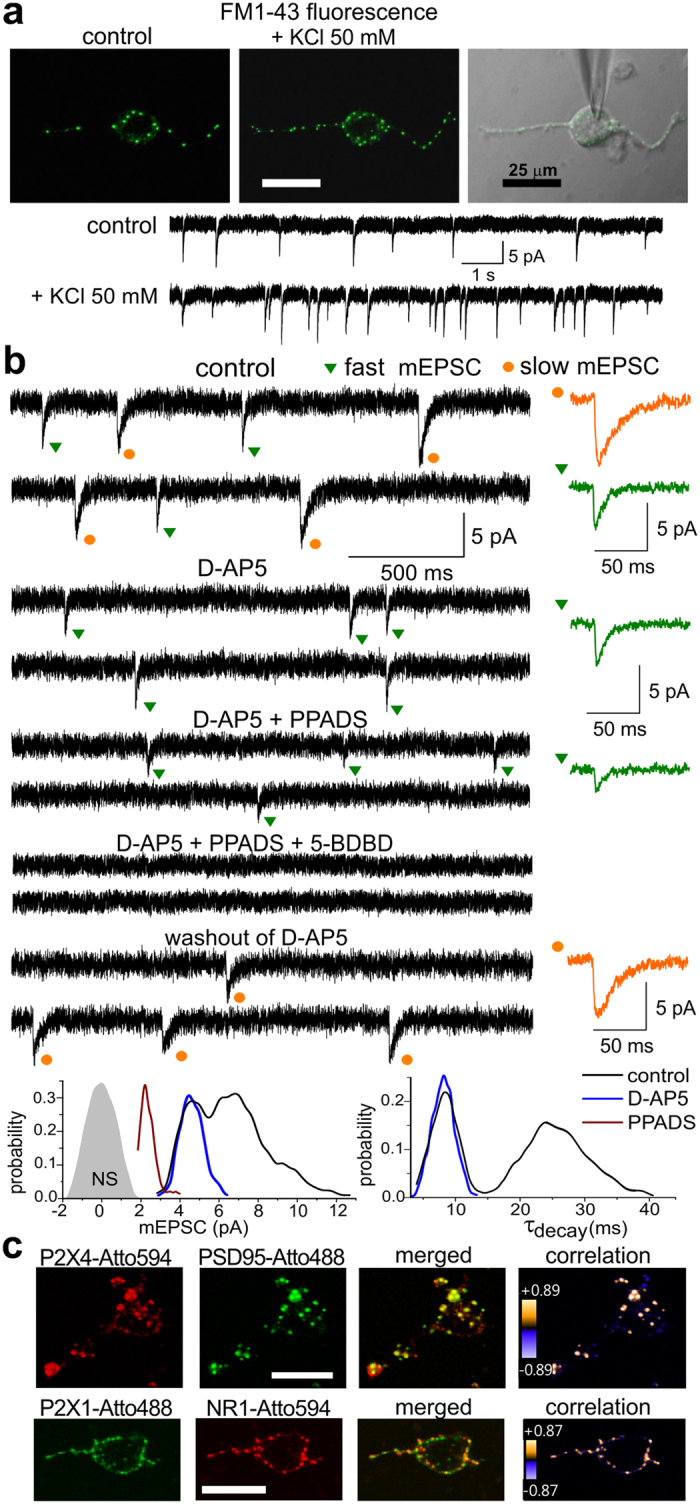
Acutely isolated neurones retain functional synaptic boutons. (**a**) Whole-cell currents were recorded in the acutely isolated neocortical layer II/III pyramidal neurone simultaneously with staining with synaptic vesicular marker FM1–43. The graphs below fluorescent and gradient contrast images of show spontaneous currents recorded at the membrane potential of −40 mV in the presence of NBQX (50 μM) and picrotoxin (100 μM). The 3 min-long application of 50 mM KCL and 2.5 μM FM1–43 induced the burst of spontaneous EPSCs accompanied by the increase in FM1–43 staining suggesting the presence of functional synapses at the dendrites and soma of acutely isolated neurone. (**b**) Example of mEPSCs recorded in the acutely isolated neocortical pyramidal neurone at −40 mV in the presence of 1 μM TTX, 100 μM picrotoxin and 50 μM NBQX (control) and after application of antagonists of NMDA and P2X receptors. The corresponding amplitude and decay time distributions (probability density functions) are shown below; shaded area shows the amplitude distribution of background noise. Dots indicate the faster (green) and slower (orange) mEPSCs. Insets on the left show the average waveforms of fast and slow synaptic currents (20 mEPSCs each). D-AP5 selectively and reversible eliminated mEPSCs with larger amplitudes and slower kinetics. P2 receptor antagonist PPADS (10 μM) significantly decreased the amplitude of fast mEPSCs, consecutive addition of 10 μM of 5-BDBD, selective antagonist of P2X4 receptors, eliminated spontaneous events. These data indicate a presence of two separate fractions of synaptic currents mediated by NMDA and P2X receptors. (**c**) Representative 2-photon images of acutely isolated neurone stained with antibodies to P2X1, P2X4 and NR1 receptor subunits and postsynaptic protein PSD95; antibodies were conjugated to fluorescent dyes Atto488 and Atto594 and applied to living neurones. P2XR and NMDAR antibodies have an extracellular epitope. The correlation between green and red fluorescence is depicted as product of the differences from the mean (PDM) for each pixel; positive values (bright yellow) are indicative for good co-localisation, negative values (blue-violet) indicate segregation.

Individual neocortical pyramidal neurones devoid of glial influence were isolated using non-enzymatic technique of vibro-dissociation which retains functional synapses on the neuronal membrane^18^ thereby providing an unique opportunity to stimulate individual synapses with efficient control of membrane voltage and extracellular milieu (Fig. 1). Spontaneous miniature currents were observed in 65 of 72 (90%) of isolated neocortical pyramidal neurones (Fig. 1a); the amplitude and kinetics of these currents resembled those of miniature synaptic currents (mEPSCs) recorded from pyramidal neurones in neocortical slices. To verify the synaptic origin of miniature currents, isolated cells were stained with the marker of synaptic vesicles FM1–43 (Fig. 1a; see also *Methods*). Activity-dependent punctate FM1–43 staining was observed in 23 of 25 cells tested. Fluorescence of FM1–43 and the frequency of miniature currents significantly increased in the presence of 50 mM KCl (Fig. 1a). High extracellular potassium depolarised presynaptic terminal (as opposed to voltage-clamped neurones) thus activating presynaptic Ca^2+^-channels and enhancing exocytosis from presynaptic terminal. Hence, spontaneous currents observed in the acutely isolated neurones most likely originated from the synaptic exocytosis of neurotransmitters.

Spontaneous events recorded at the membrane potential of −40 mV in the presence of 1 μM TTX, 50 μM NBQX and 100 μM picrotoxin (Fig. 1b) had the average frequency of 0.18 ± 0.07 Hz and average amplitude of 5.8 ± 3.9 pA (n = 47). Distributions of amplitude and decay time of these mEPSCs showed two distinct populations (Fig. 1b), which could be separated pharmacologically with NMDAR and P2XR antagonists. The first population (~62%) of mEPSCs exhibited slower kinetics with average decay time of 24.7 ± 6.6 ms (n = 47) and large amplitude (6.7 ± 2.6 pA, n = 47). These mEPSCs were inhibited by NMDAR antagonist D-AP5 (30 μM) in all 15 neurones tested. The second, non-glutamatergic fraction of mEPSCs had average decay time of 9.2 ± 0.9 ms and average amplitude 4.3 ± 2.1 pA (n = 47). The amplitude of the faster currents was reduced by purinoreceptor antagonists PPADS (10 μM) by 52 ± 14% (n = 15, Fig. 1b). Incomplete inhibition probably reflects contribution of P2X_4_Rs insensitive to PPADS. Application of a specific P2X_4_R antagonist 5-BDBD together with PPADS completely eliminated non-glutamatergic mEPSCs (n = 7). These data show that synaptic release of ATP can activate distinct excitatory synaptic currents mediated by P2XRs of several sub-types, in particular P2X_4_Rs.

The presence of postsynaptic P2XRs was also supported by immunostaining of living neurones (Fig. 1c). Immunolabelling of isolated pyramidal neurones with antibodies to P2X_4_Rs showed punctate staining co-localized with PSD95 protein (Fig. 1c). The intensity correlation quotient (ICQ) and Pearson’s correlation coefficient for anti-P2X_4_ vs. anti-PSD95 staining (see *Methods*) were, respectively, 0.83 ± 0.07 and 0.44 ± 0.06 (n = 7) indicating a good correlation. The immunostaining of pyramidal neurones with anti-P2X_1_R antibodies also showed punctate pattern and good correlation with anti-GluN1 staining in all 8 cells tested.

**Figure 2:**
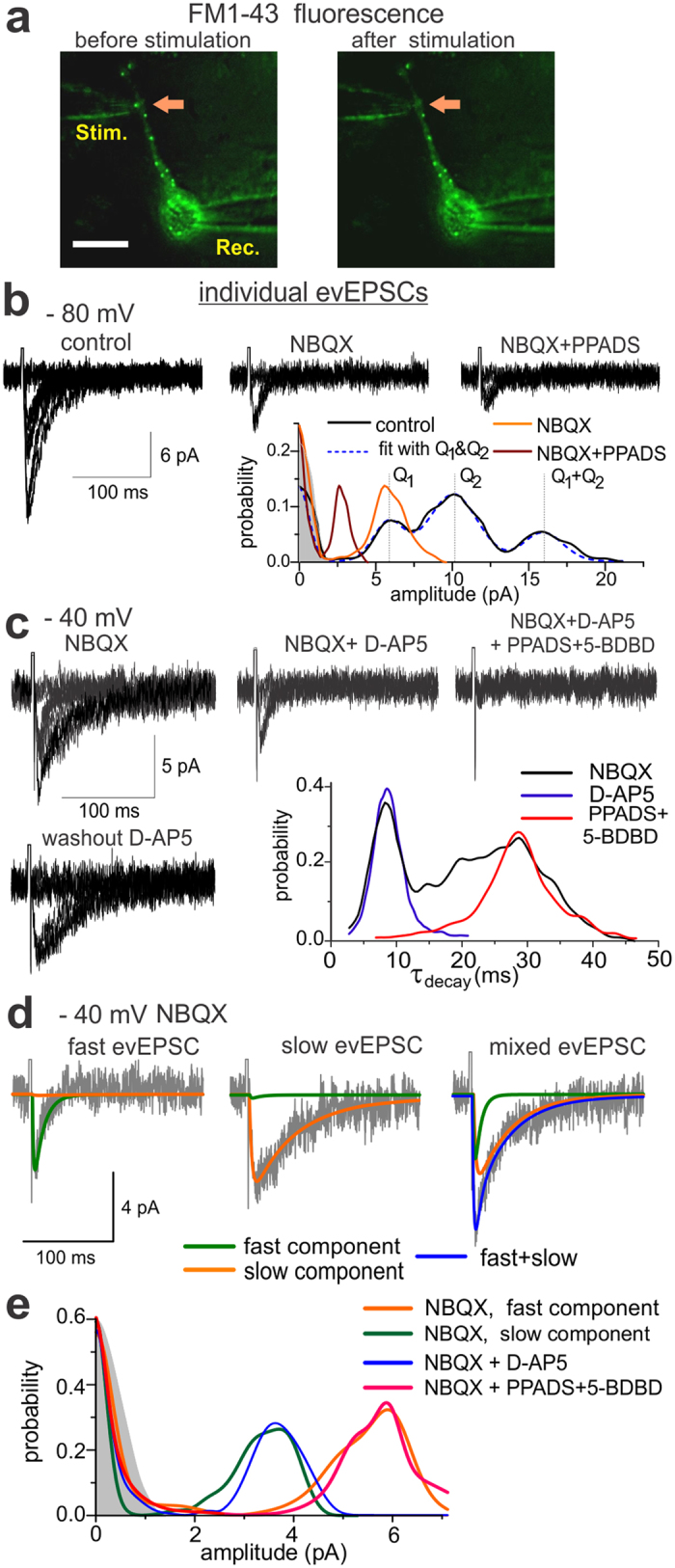
Stimulation of individual neocortical synapses elicits glutamateric and purinergic synaptic currents. (**a**) Extracellular electric field stimulation of synaptic bouton was identified based on FM1–43 fluorescent and gradient contrast imaging (superimposed). Note the decrease in the FM1–43 fluorescence after repetitive stimulation supporting the exocytotic origin of evoked responses. (**b**–**e**) Electric field stimulation of synaptic bouton evoked quantal fluctuating currents which were mediated by glutamate and ATP receptors. (**b**) Each panel shows 10 consecutive single-bouton evoked currents (evEPSCs) recorded at −80 mV in presence of 100 μM picrotoxin before (control) and after application of 50 μM NBQX and 10 μM PPADS. Lower panel shows the corresponding amplitude distributions and theoretical compound binomial distribution with two qunatal sizes (best fit using maximum likelihood method^40^); shaded area shows the amplitude distribution of background noise. Inhibition of AMPA receptors reveals residual quantal EPSCs sensitive to specific P2 receptor antagonist PPADS. Purinergic and glutamatergic single-bouton evPSCs have different quantal sizes, Q_1_ and Q_2_, and can be evoked simultaneously (amplitude distribution peak at Q_1_ + Q_2_). (**c**,**d**) single-bouton evEPSCs recorded at −40 mV in presence of NBQX and picrotoxin exhibited diverse kinetics. (**c**) Individual evEPSCs recorded in control (NBQX and picrotoxin), after application of D-AP5 (30 μM) and consecutive application of P2X receptor antagonists PPADS (10 μM) and 5-BDBD (10 μM) and washout of D-AP5. Lower left panel shows the corresponding distributions of decay time of non-zero responses, determined by the best fit with single exponential curve. Inhibition of NMDA receptors selectively and reversibly eliminated evEPSCs of slower kinetics whereas inhibition of P2X receptor affected the fast responses. (**d**) Separation of fast and slow components of control evEPSC. Blue lines show the best fit of individual evEPSCs as a sum of fast (green) and slow (orange) components. (**e**) amplitude distributions for fast and slow components of control evEPSCs and the whole evEPSCs recorded in presence of D-AP5 or PPADS plus 5-BDBD. Note the good correspondence between purinergic evEPSCs (blue) and fast component of control evEPSCs (green) and between glutamatergic evEPSCs (red) and slow component of control evEPSCs (orange).

The co-localisation of P2X and NMDA receptors suggests that purinergic and glutamatergic mEPSCs may appear in the same synapses. Still, judging from the electrophysiological recordings, they occurred asynchronously (Fig. 1b,c). This may reflect ATP release from the separate pool of vesicles sharing the same terminals with glutamate-containing vesicles. To verify this, we investigated the synaptic responses elicited in the individual neocortical synapses (Fig. 2a). Synapses (visualised with FM1–43) were activated by electric field stimulation delivered via a glass pipette positioned in a close proximity to an identified synaptic bouton (see *Methods* and ref. 19). Stimulation of single synapse evoked fluctuating quantal currents with significant number of zero responses thus supporting their synaptic origin (Fig. 2b). Repeated stimulation caused rapid decrease in FM1–43 fluorescence only in the tested synapse verifying the exocytotic nature of the response (Fig. 2a). Similarly to previous experiments^19^, responses could be enhanced by increasing stimulus duration from 1 to 5 ms (Fig. S1), and were inhibited by removal of extracellular Ca^2+^ (Fig. S1), indicating that neurotransmitter release was triggered via presynaptic Ca^2+^-channels directly activated by depolarisation of the presynaptic membrane^19^.

Synaptic responses were recorded from 62 out of 98 synaptic boutons, located on the dendrites of pyramidal neurones (33 cells tested), at −80 mV and in the presence of picrotoxin (100 μM). Application of 50 μM NBQX revealed the non-glutamatergic evoked EPSCs (evEPSCs) in 38 out of 62 synapses (Fig. 2b). The residual single-bouton evEPSCs were inhibited by PPADS (by 53 ± 15%, n = 12) and completely eliminated by mixture of PPADS and 5-BDBD (Fig. 2b,c). In 33 synaptic boutons, exhibiting both glutamatergic and non-glutamatergic currents, evEPSCs recorded at −40 mV were sensitive to NMDAR-antagonists (Fig. 2c). When AMPARs, NMDARs and P2XRs blockers were applied in combination, only zero responses were recorded (the last graph in the Fig. 2c). The P2XR-mediated quantal currents could also be detected without inhibition of glutamatergic evEPSCs, in this case purinergic currents could be distinguished by their different quantal size as a separate peak in the amplitude distribution (Fig. 2b). In the majority of synaptic boutons tested, we could also observe a superposition of P2X and AMPA receptor-mediated quantal currents. This was evidenced by amplitude distributions exhibiting three clear peaks (Fig. 2b), corresponding to P2XR-mediated and AMPAR-mediated quantal currents and their superposition. We observed a similar quantal pattern in distributions of single-axon evEPSCs in our previous experiments in the neocortical neurones in slices^17^. These results verify the possibility of co-release of ATP and glutamate in the same synaptic terminal and contribution of P2XRs to synaptic signalling in the significant fraction of glutamatergic synapses.

We also observed significant enhancement of P2XR-mediated currents after 10–20 min of stimulation (Fig. S2) which was inhibited by intracellular perfusion with compounds affecting endocytosis and trafficking of P2XRs^20^,^21^,^22^. This agrees with previous data on activity-dependent trafficking and mobility of P2X receptors^20^. The activity-dependent recruitment of P2XRs can explain the relatively high probability of detection of purinergic EPSCs in a single synapse, because purinergic currents were usually recorded after 15–20 min of bouton stimulation.

Stimulation of individual boutons in the presence of NBQX (Fig. 2c) revealed two distinct populations of responses. The largest fraction (45%) of evEPSCs had average decay time of 27.8 ± 7.9 ms and was inhibited by D-AP5 in all 22 boutons tested. Significant fraction (29%) of evEPSCs showed much faster kinetics with decay time of 9.6 ± 1.5 ms; these currents were eliminated by application of PPADS and 5-BDBD (n = 7). The remaining 26% of evEPSCs exhibited mixed kinetics. The difference in the kinetics of P2XR and NMDAR-mediated currents allowed to resolve their amplitude distributions. Each evEPSC, recorded in presence of NBQX, was fitted with the sum of the fast and slow theoretical waveforms (Fig. 2d, see also *Methods*). Both fast and slow components demonstrated a quantal behaviour (Fig. 2d). The amplitude distribution of the fast component of evEPSC did not differ significantly from the pure purinergic evEPSC recorded in the presence of NBQX and D-AP5 (Fig. 2e) in all 15 boutons tested. Conversely, amplitude distribution of the slow evEPSCs did not differ from NMDAR-mediated evEPSCs recorded after blocking of P2X receptors (n = 7). The quantal size of fast purinergic evEPSCs was 3.9 ± 1.5 pA (n = 15); the quantal size of slow D-AP5-sensitive currents was 6.2 ± 1.8 pA (n = 15). These values were consistent with unitary amplitudes of P2XR- and NMDAR-mediated spontaneous mEPSCs (Fig. 1a). Combined, these data demonstrate that excitatory neocortical synapses harbour functional postsynaptic P2XRs, which can be activated by ATP co-released with glutamate from the excitatory nerve terminals. The co-localisation of P2X and NMDA receptors may facilitate their interaction, which we tested in the following experiments.

### P2X receptors cause down-regulation of NMDA receptors in the individual synapses via Ca^2+^-dependent mechanism

**Figure 3:**
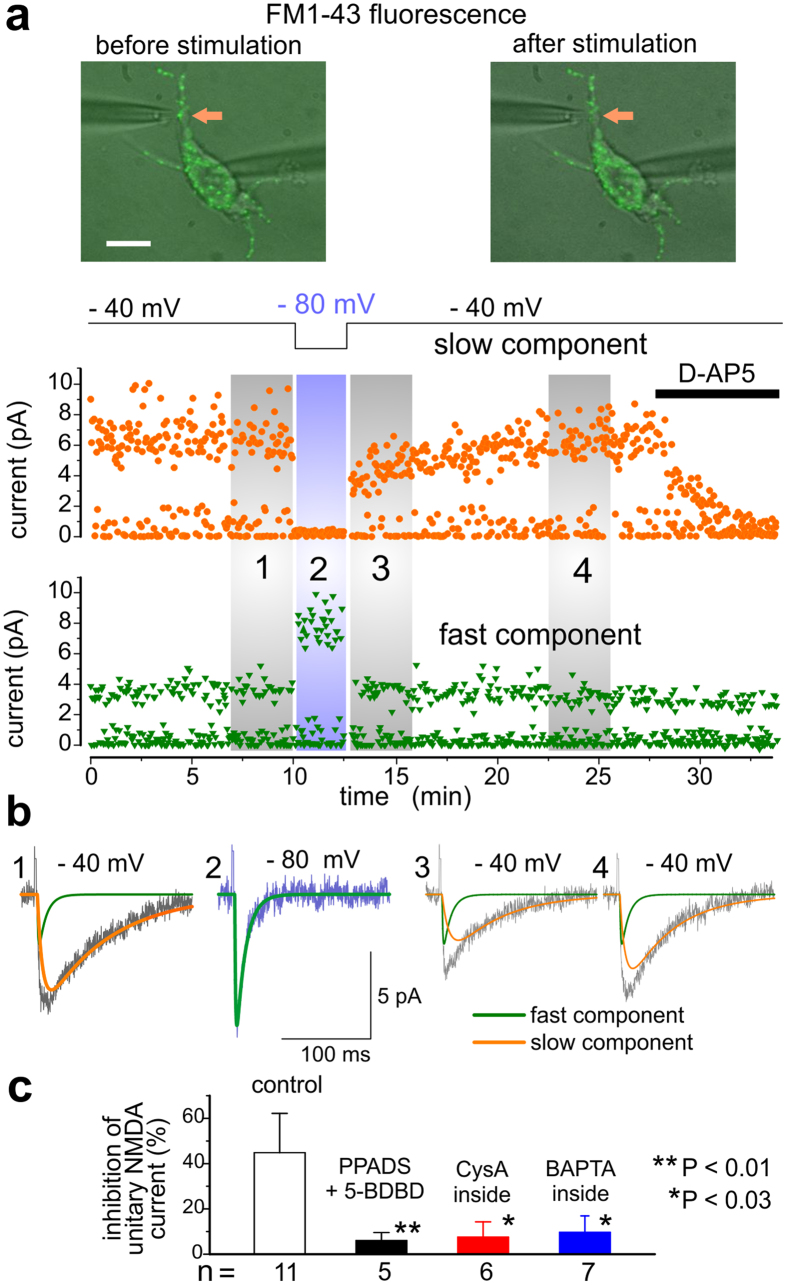
Inactivation of single-bouton NMDA EPSCs after series of purinergic responses. (**a**) Individual synaptic boutons on the dentrites of isolated neurones were identified and stimulated as shown in the Fig. 2. Single-bouton evEPSCs were recorded in presence of 50 μM NBQX and 100 μM picrotoxin at membrane potentials of −40 mV and −80 mV, as shown in the top graph. Middle and bottom graphs in (A) show time course of changes in the amplitude of fast and slow components of evEPCS, determined as shown in Fig. 2d. Each dot represents single synaptic current. (**b**) The corresponding examples of evEPSCs (average of 20 consecutive responses) recorded at moments indicated in the panel (**a**). Green and orange line show correspondingly fast and slow components. Stimulation of bouton at −80 mV evoked only the fast component, stimulation at −40 mV evoked both fast and slow components, slow component was selectively eliminated by D-AP5 (30 μM). The slow and fast components were attributed correspondingly to the NMDA and P2X receptors, as shown in the Fig. 2. EPSCs were stimulated at 0.33 Hz at +40 mV and at 1 Hz at −80 mV. (**a**,**b**) Prolonged period of activation of P2X receptors (region 2) led to reduction in the amplitude of NMDA receptor-mediated component (region 3) which gradually recovered after then. (**c**) Pooled data on reduction of the single-bouton NMDA-receptor mediated current, recorded using the above protocol in the control and in presence of extracellular PPADS (10 μM) and intracellular cylsosporin A (500 nM) or BAPTA (3 mM). The decrease in the NMDA receptor-mediated currents was evaluated by comparison of mean amplitude of slow component of evEPSCs 5 min before (region 1) and during first 4 min after P2X receptors stimulation (regions 3). Data are shown as mean ± SD for number of experiments as indicated. Asterisks (*) and (**) Indicated statistical significance of difference in the NMDA current reduction from the control. Note that inhibition of NMDA currents was significantly attenuated by the antagonist of P2 receptors, blocker of phosphatase 2B and Ca^2+^ chelator.

**Figure 4:**
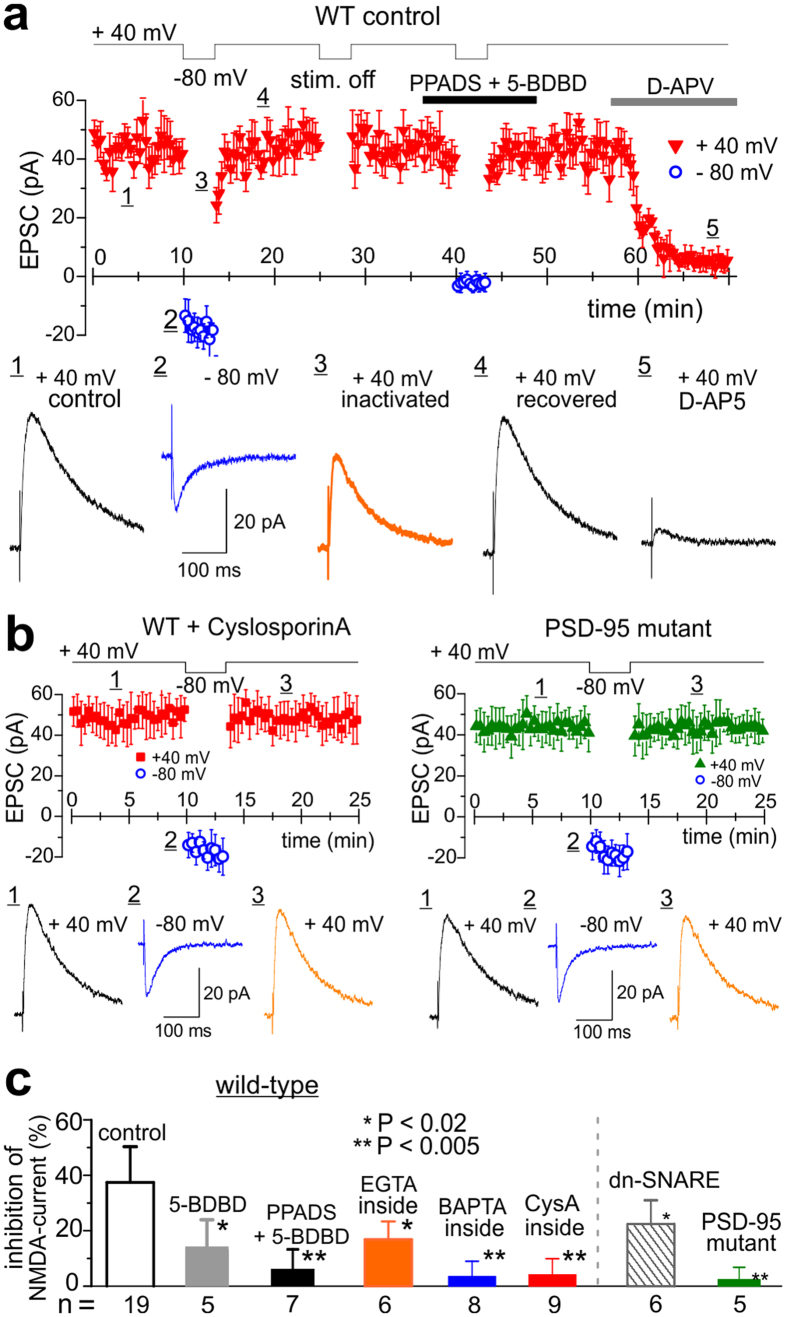
Interaction between NMDA and P2X receptors requires PSD-95 complex. Inactivation of NMDA EPSC after series of purinergic responses was measured in the pyramidal neurones of neocortical slice of wild-type and PSD-95 mutant mice. (**a**) Synaptic currents were recorded in the pyramidal neurones of cortical layer II/III in the presence of 50 μM NBQX, 100 μM picrotoxin and 10 μM DCPPX at membrane potentials of +40 mV and −80 mV, as shown in the top graph. Neurone were perfused with intracellular solution containing either 0.1 mM EGTA (control), 10 mM EGTA, 3 mM BAPTA or 500 nM cyclosporine A. EPSCs were stimulated at 0.2 Hz at +40 mV and at 1 Hz at −80 mV. Middle graph shows the time course of changes in the amplitude EPCS; each dot represents mean ± SD for EPSCs recoded within 30 sec-long period. Lower panel shows the corresponding examples of EPSCs (average of 20 responses) recorded at the moments indicated. Stimulation of EPSCs at −80 mV evoked the P2X-receptor-mediated current as evidenced by inhibition with selective antagonists PPADS (10 μM) and 5-BDBD (5 μM). EPSCs stimulated at +40 mV were mediated mainly by NMDA receptors as evidenced by inhibition with D-AP5 (30 μM). Note the substantial reduction in the magnitude of outward NMDA receptor-mediated EPSCs recorded after the episode of stimulation of purinergic EPSCs at −80 mV. Switching membrane potential without stimulation did not have any effect. Reduction of outward NMDAR EPSCs was attenuated by inhibition of P2X receptors. (**b**) EPSCs were recorded using similar protocol in the neurone of wild-type mouse perfused with cyclosporin A (500 nM) and neurone of PSD-95 mutant mouse in control conditions. (**c**) Pooled data on the reduction of the outward NMDAR-mediated current, recorded using the above protocol in the wild-type, dn-SNARE and PSD95 mutant mice. Data are shown as mean ± SD for number of experiments as indicated. Note that inhibition of NMDA currents was significantly inhibited in the PSD-95 mutants and in the wild-type mice by application of the antagonist of P2 receptors or intracellular perfusion with blocker of PP2B and Ca^2+^ chelators.

As alluded above (Fig. 2), P2XR- and NMDAR-mediated currents elicited in the same synapse can be separated by their decay kinetics into fast and slow components respectively. To dissect the specific contribution of P2X receptors in the modulation of NMDAR-mediated component of EPSC, we exploited their different voltage-dependence (Fig. 2b,c). We recorded baseline evEPSCs at −40 mV in presence of NBQX and then switched the membrane potential to −80 mV for 5 min (Fig. 3a) when single-bouton currents were mediated solely by the P2X receptors (region 2 in Fig. 3a). Slow NMDAR-mediated evEPSCs, recorded immediately after returning of the membrane potential to −40 mV (Fig. 3a, region 3) were significantly reduced (Fig. 3b,c). Subsequently, the amplitude of slow component of synaptic response gradually recovered. To evaluate the effect of down-regulation of NMDA receptor-mediated currents, we compared the mean amplitude of slow component of evEPSCs 5 min before and 5 min after P2X receptors facilitation (regions 1 and 3 in Fig. 3a). The decrease in the NMDAR-mediated component of synaptic response was 44.8 ± 17.5% (n = 11) under control conditions but reached only 19.3 ± 10.5% (n = 7) when P2XRs were inhibited (Fig. 4c). The intracellular perfusion of neurones with phosphatase 2B inhibitor cyclosporine A (500 nM) or BAPTA (3 mM) strongly reduced the down-regulation of NMDAR-mediated currents (Fig. 3c) suggesting an involvement of postsynaptic Ca^2+^-elevation and de-phosphorylation. The cross-talk between agonist-elicited P2XR and NMDAR currents in the isolated neocortical neurones exhibited similar properties, in particular dependence on the influx of Ca^2+^ via P2XRs (Fig. S3).

Similarly to evoked single-bouton currents, spontaneous NMDAR-mediated mEPSCs were significantly reduced after 5 min-long application of P2X receptors agonists α,β-meATP and ATP in all of 15 neurones tested (Fig. S4a,b). Intracellular perfusion of neurones with BAPTA (3 mM) or cyclosporine A (500 nM) prevented the inhibition of NMDAR currents (Fig. S4c). There was no reduction in the frequency of slow mEPSCs (Fig. S4b) whilst their quantal amplitude was significantly decreased (Fig. S4c). These data strongly support the postsynaptic mechanism of inhibition of slow mEPSCs.

Hence, co-release of ATP and glutamate in the same excitatory synapse can down-regulate NMDA receptors via a postsynaptic Ca^2+^-dependent mechanism.

### Interaction between purinoreceptors and NMDA receptors involves PSD-95 complex

The data presented above suggest (similarly to previous reports^23^,^24^) the role for phosphatase 2B (PP2B) in the purinergic modulation of postsynaptic NMDA receptors. Proteomic and functional analyses of NMDA receptor complexes show that PP2B can be physically associated with NMDA receptor via scaffold protein AKAP5 and PSD-95 and can play important role in the regulation of NMDARs by the PSD-95 multi-protein complex^25^,^26^,^27^. We compared the purinergic modulation of NMDARs in the pyramidal neurones in neocortical slices of the wild-type and PSD-95 mutant mice^28^. Excitatory postsynaptic currents (EPSCs) were recorded from pyramidal neurones of cortical layer II/III in the presence of 10 μM DCPPX, 50 μM DNQX, 100 μM picrotoxin at the membrane potential of +40 mV (to access the NMDAR contribution, sensitive to D-APV, Fig. 4a) and −80 mV (to isolate P2XR component, sensitive to PPADS and BDBD, Fig. 4a). The NMDAR-mediated EPSCs were significantly suppressed immediately after activation of P2X receptors at −80 mV (region 3 in Fig. 4a) and gradually recovered afterwards (region 4 on Fig. 4a). Inhibition of NMDAR-mediated currents was attenuated by intracellular cyclosporine A (500 nM), EGTA (10 mM) and BAPTA (3 mM) and was abolished by purinoreceptor antagonists PPADS (10 μM) and 5-BDBD (5 μM). In PSD-95 KO mice, the inhibition of NMDAR-mediated currents did not occur even under control conditions (Fig. 4b). These results indicate that Ca^2+^ influx via P2X purinoreceptors can down-regulate NMDA receptors via phosphatase 2B associated with PSD-95 complexes (most likely via AKAP5^27^).

In brain slices, stimulation of synaptic release of neurotransmitters can evoke Ca^2+^-signals in astrocytes and thereby trigger release of ATP^2^,^3^. To test the possible involvement of this pathway, we used the dn-SNARE transgenic mice with inducible and selective impairment of astrocytic exocytosis^29^,^30^. Down-regulation of NMDAR-mediated currents was significantly reduced in the dn-SNARE mice (Fig. 4b), suggesting the role for astroglia in modulation of synaptic NMDARs.

The above data suggested that transient elevation of ATP concentration in the brain tissue could cause the P2XR-mediated down-regulation of NMDA receptors, putatively working as negative feedback limiting the consequences of glutamate excitotoxicity. This notion was supported by experiments with transient bath application of 10 μM ATP or α,β-meATP to neocortical slices (Fig. S4). Similar individual synapses on isolated neurones (Fig. S3), both ATP and α,β-meATP caused significant reduction in the amplitude of NMDAR-mediated component of EPSCs evoked in pyramidal neurones *in situ* (Fig. S4a,c). Since we observed the simultaneous moderate increase in the amplitude of AMPAR-mediated component (Fig. S4a), the reduction of NMDAR-mediated EPSCs could hardly be attributed to pre-synaptic decrease in the release of glutamate. Involvement of postsynaptic Ca^2+^-dependent mechanism was also supported by attenuation of the purinergic modulation by intracellular Ca^2+^-chelators and PP2B inhibitor (Fig. S4c). Interestingly, the up-regulation of AMPAR-mediated synaptic current was not affected by PP2B inhibitor (Fig. S4b) suggesting that purinergic modulation of AMPA receptors in neocortex might involve mechanisms, different from AKAP5-PP2B pathway recently found in the hippocampal neurones^31^,^32^.

### Release of ATP from glial cells is crucial for interaction between purinoceptors and NMDA receptors *in situ*

Our previous results suggest that ATP released from astrocytes can activate P2X receptors at the periphery of synaptic spine^3^. Consequently, glia-derived ATP could directly modulate postsynaptic NMDARs. Alternatively, activation of extrasynaptic P2XRs by glia-derived ATP could promote their mobility and recruitment to synapses thus enhancing the effect of synaptic release of ATP. To explore the role for ATP originated from glia in more details, we employed two experimental approaches: (1) recording of mEPSCs in presence of TTX to minimize the involvement of action potential-dependent synaptic release of ATP and (2) recording of single-axon synaptic currents which are not accompanied by Ca^2+^-elevation in astrocytes (Fig. S6).

**Figure 5:**
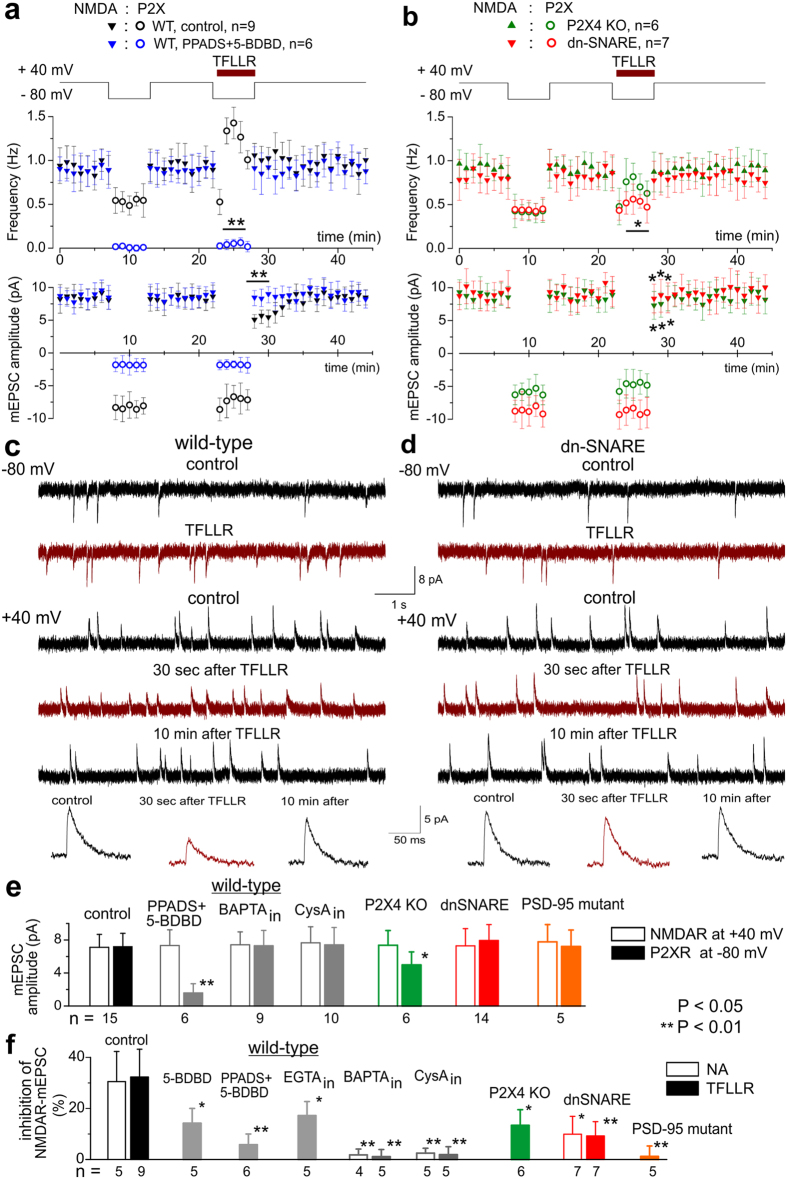
Activation of astroglial exocytosis down-regulates neuronal NMDA receptors via P2X receptor-dependent mechanism. Release of ATP from astrocytes was activated via PAR1 or α1-noaradrenaline receptors^3^,^4^. Spontaneous synaptic currents were recorded from neocortical pyramidal neurones of wild-type mice (**a**,**c**) and dn-SNARE and P2X_4_R KO mice (**b**,**d**) in presence of DNQX (50 μM) and picrotoxin (100 μM) as shown in the upper graph^44^,^45^. The mEPSCs recorded at −80 mV were mediated solely by P2XRs, as verified by inhibition with PPADS and 5-BDBD. The NMDAR-mediated currents were measured at +40 mV; they were distinguished from P2XR-mediated mEPSCs by their slower kinetics and larger quantal amplitude as shown in Figs 1 and 2. (**a**,**b**) Changes in the P2X and NMDA receptor-mediated mEPSCs after application of PAR1 receptor agonist TFLLR (10 μM). Each dot represents mean ± SD for mEPSCs recorded within 1 min-long period and pooled for number of experiments indicated. Asterisks (*) indicate statistically significant difference between wild-type and transgenic mice, P < 0.05. Asterisks (**) indicate statistical significance of the effect of PPADS and 5-BDBD in the wild-type mice, P < 0.01. Note that application of TFLLR caused the significant increase in the frequency of P2X receptor-mediated events in the wild-type mice. The TFLLR-induced burst of purinergic events were strongly reduced in the P2X4 KO and the dn-SNARE mice. (**c**,**d**) The representative whole-cell currents and average mEPSCs waveforms (20 events each) recorded in the neurones of wild-type and dn-SNARE mice as indicated. (**e**) Pooled data on the baseline amplitude of outward NMDAR-mediated mEPSCs and inward P2XR-mediated mEPSCs under different conditions. (**f**) The relative reduction of the outward NMDAR-mEPSCs after activation of astrocytes by noradrenalin (NA) and TFLLR. Data are shown as mean ± SD for number of experiments indicated. Note the significant decrease in the amplitude of NMDAR-mediated outward mEPSCs recorded in the wild-type mice within 4 min after application of TFLLR and NA. Glia-induced down-regulation of NMDA receptors was abolished by P2X receptors antagonists and intracellular BAPTA or PP2B inhibitor and was significantly attenuated in P2X_4_R KO and dn-SNARE mice whereas the basal amplitude of mEPSCs was not significantly affected.

First, we monitored spontaneous NMDAR-mediated currents at +40 mV and P2X-mediated currents at −80 mV (Fig. 5). Selective activation of astrocytes was achieved by stimulation of PAR-1 and α1-noradrenaline receptors, which induce Ca^2+^-signals in astrocytes but not in neurones^3^,^4^. To verify the astroglial origin of observed effects, we used the dn-SNARE mice; involvement of neuronal P2X receptors was confirmed by usage of P2X_4_R knock-out mice^3^,^33^. The NMDAR-mediated mEPSCs were segregated by their slower decay time and larger quantal size, similar to single-bouton currents (Figs 1 and 2).

Activation of astrocytes was accompanied by an increase in the frequency of P2XR-mediated currents (Fig. 5a), majority of which had slower kinetics and smaller amplitudes (Fig. 5c). As in our previous studies^3^,^4^, the frequency of these currents was much less in the dn-SNARE and in P2X_4_ KO mice (Fig. 5b,d) indicating that the burst of purinergic activity was due to exocytosis of ATP from astrocytes. The amplitude of NMDAR -mediated mEPSCs was significantly reduced (by 28.7 ± 9.5%, n = 9) during first 3 minutes after the glia-driven activation of purinergic currents (Fig. 5a,c). Application of 1 μM noradrenaline (NA) elicited the burst of purinergic activity followed by 30.4 ± 10.8% (n = 8) decrease in the amplitude of NMDAR-mediated currents (Fig. 5e).

Recording of spontaneous purinergic currents at −80 mV without activation of astrocytes did not produce similar effect (Fig. 5a, first period of −80 mV). We did not observe any decrease in the frequency of glutamatergic mEPSCs, which argues against the presynaptic mechanism of depression of NMDAR-mediated currents.

To verify that astrocyte-induced modulation of NMDARs involves the PP2B-PSD-95 cascade, we used similar experimental tools as described above (Fig. 4). Intracellular perfusion of neurones with BAPTA or cyclosporine A did not cause notable changes in the baseline amplitude of NMDAR-mediated and P2XR-mediated mEPSCs (Fig. 5e) but strongly attenuated the TFLLR- and NA-triggered inactivation of NMDAR-mediated currents (Fig. 5f). The reduction of NMDA currents was also abolished by inhibition of P2XRs with PPADS and 5-BDBD and was decreased in the P2X_4_R KO and in PSD-95 KO mice (Fig. 5b,f). The TFLLR- and NA-triggered inactivation of NMDARs was significantly less in the dn-SNARE mice (Fig. 5f) again supporting the role of glial exocytosis.

**Figure 6:**
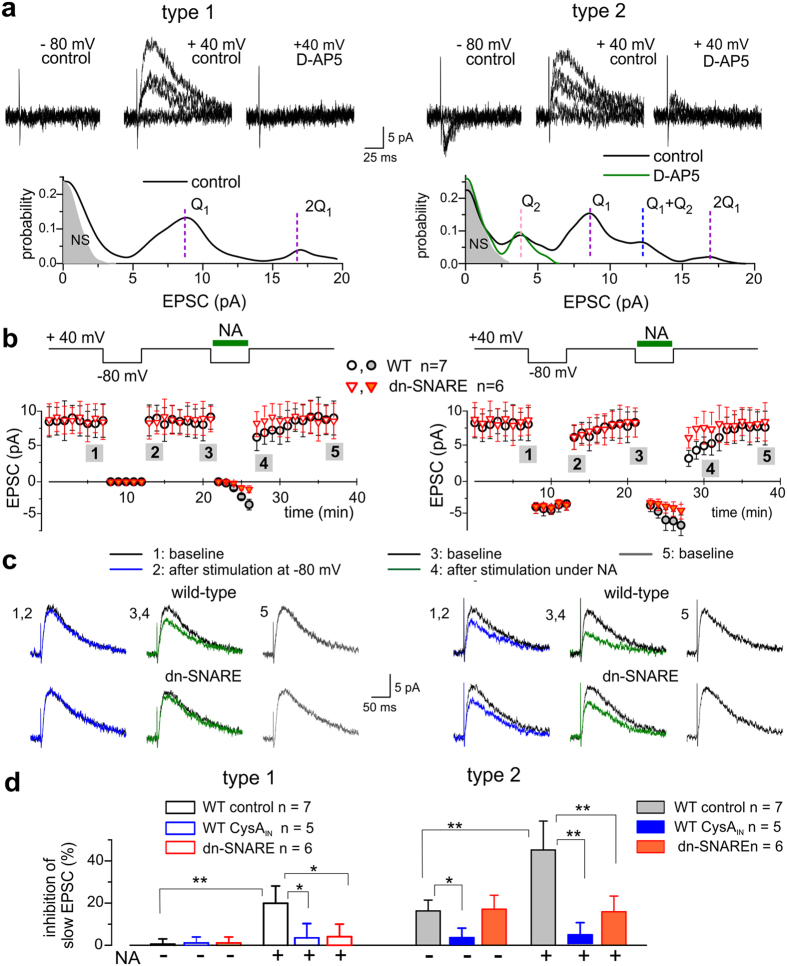
Inactivation of NMDA receptors in the individual neocortical synapses *in situ*. The single-axon P2XR and NMDAR-mediated EPSCs were evoked by minimal stimulation in the layer 2/3 pyramidal neurones of wild-type and dn-SNARE mice. The panels (**a**–**c**) show examples of two type of synapses correspondingly lacking (type 1, left) and exhibiting (type 2, right) the P2XR-mediated EPSCs. (**a**) Representative EPSCs recorded at −80 mV and +40 mV in baseline conditions (100 μM picrotoxin and 50 μM DNQX) and in the presence of 30 μM D-AP5. Lower panels show amplitude distributions of EPSC at +40 mV. Note the presence of muliti-quantal NMDAR-mediated currents in both type 1 and 2 synapses (verifying the efficiency of stimulation) and presence of quantal non-glutamatergic current only in the type 2 synapse. In the type 2 synapse, quantal NMDAR- and P2XR-mediated currents (Q_1_ and Q_2_,) can be evoked simultaneously (amplitude distribution peak at Q_1_ + Q_2_). (**b**–**d**) Inactivation of NMDA receptors was induced by episode of 1 Hz stimulation at −80 mV (similar to Figs 4 and 5) in the baseline conditions and in presence of 3 μM noradrenaline. (**b**) Time course of the mean EPSC amplitude; each dot represents mean ± SD for EPSCs recorded within 1 min-long period and pooled for number of experiments indicated. (**c**) The average EPSCs waveforms (20 responses each) recorded at +40 mV as indicated in (**b**): 1,2 – before stimulation at −80 mV; 3,4 – 1 min after; 5 – recovery. (**d**) Pooled data (mean ± SD for number of experiments indicated) on the reduction of the slow component of outward NMDAR-mediated EPSCs in control and in neurones perfused with cyclosporine A. Asterisks (*) and (**) Indicate statistically significant difference with P < 0.05 and P < 0.01 correspondingly. Note the lack of inhibition of NMDAR-mediated currents in the type 1 synapses at baseline conditions and moderate effect in the presence of NA. In the type 2 synapses, effect was moderate in the baseline conditions and increased after application of NA in WT but not in the dn-SNARE mice. Inhibition of PP2B abolished the effect of NA.

In the second set of experiments (Fig. 6), we used the minimal stimulation barely exceeding the threshold of generation of single-axon synaptic current^17^ (see also *Methods*). This protocol does not activate Ca^2+^-elevation in astrocytes (Fig. S6), so any observed effects on NMDAR-currents would originate solely from synaptic release of ATP. Similarly to our previous observations^17^, two types of behaviour of single-axon EPSCs recorded in the presence of AMPA receptor antagonists were observed (Fig. 6a). In 58% of neurones tested, no response to minimal stimulation at −80 mV was detected, whereas at +40 mV we observed quantal NMDAR-mediated currents (type 1 response). In other 42% of cells, we observed the fast quantal EPSCs at −80 mV and fast and slow EPSCs at +40 mV; fast EPSCs were fully abolished by application of the P2X antagonists (type 2 response).

Similarly to experiments described above (Figs 3 and 4), we triggered the inactivation of NMDAR-mediated currents by short episode of medium-frequency (1 Hz) stimulation of purinergic currents at −80 mV (Fig. 6b). In type 1 cells lacking the P2XR-mediated currents no inactivation of NMDAR-mediated single-axon EPSCs was observed. In the type 2 neurones we observed moderate (16.3 ± 5.1%, n = 7, P = 0.04) decrease in the amplitude of slow NMDAR-mediated component of EPSCs (Fig. 6b). Although these data are in general agreement with results obtained in the single-bouton and slice experiments, the extent of down-regulation of NMDARs by single-axon purinergic current was smaller (cf. Figs 4 and 5). Inactivation of NMDARs in single boutons, however, was evaluated in conditions when the synaptic density of P2XRs was enhanced by prolonged stimulation (Fig. S2). As for the slice experiments, purinergic modulation of NMADRs could be amplified by synergistic action of glia-derived ATP.

To test the contribution of astroglia, we measured the down-regulation of single-axon NMDAR-currents when single-axon stimulation of purinergic EPCSs at –80 mV was paired with selective activation of astrocytes by stimulation of α1-adrenoceptors (Fig. 6c). In this case, down-regulation of NMDAR-mediated EPSCs was significantly potentiated both in the type 1 and type 2 neurones. The NA-induced facilitation of purinergic modulation was observed only in the wild-type but not in the dn-SNARE mice (Fig. 6c,d); it was also inhibited by the intracellular cyclosporine A (Fig. 6d).

**Figure 7:**
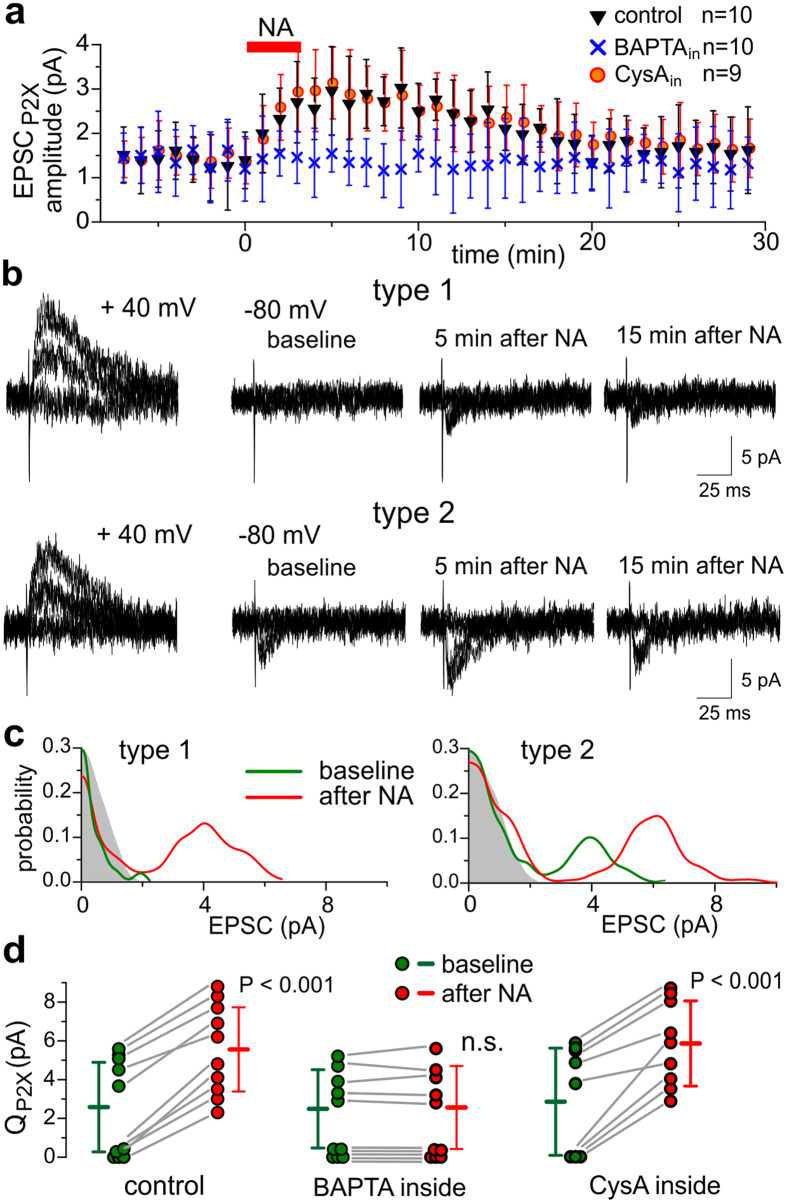
Activation of astrocytes facilitates the recruitment of P2X receptors into neocortical synapses. (**a**,**b**) The time course of average amplitude and representative individual EPSCs evoked in the neocortical neurones at −80 mV by the minimal stimulation in presence of 100 μM picrotoxin and 50 μM DNQX. Astrocytes were activated by application of 3 μM noradrenaline. (**a**) Each dot represents mean ± SD for EPSCs recorded within 1 min-long period and pooled for 10 neurones perfused with conventional intracellular solution containing either 0.1 mM EGTA (control) or 3 mM BAPTA or 500 nM cyclosporine A. Note the slow transient increase in the EPSC amplitude caused by NA. (**b**) examples of type I and type II responses, correspondingly lacking and exhibiting the P2XR-mediated component under the baseline conditions. In each neurone, the efficiency of stimulation was verified by observing the NMDAR-mediated responses at +40 mV. Presence of P2XR-mediated currents at −80 mV was verified at the end of experiment by inhibition with PPADS and 5-BDBD. In the type I neurons, new quantal purinergic currents appeared after application of NA whereas the type II neurones exhibited the marked increase in the P2X-mediated current. (**c**) Quantal behaviour of EPSC recorded at −80 mV. The graphs show the amplitude distributions of EPSCs recoded before and 4–8 min after application of NA for the neurones shown in (**b**); shaded areas show the distribution of background noise. In baseline conditions, the type 1 neurone shows only noise in the amplitude distribution. However, quantal currents appeared after application of NA. In the type 2 neurone, the quantal size increased after NA causing the rightward shift of the peak in the amplitude histogram (**c**). (**d**) Pooled data on the NA-induced changes in the quantal size of purinergic EPSCs in the control neurones and neurones perfused with BAPTA and cyclosporine A. Dots indicate individual neurones, the mean ± SD values are indicated with bars. The NA-induce changes from the baseline values were statistically significant in the control (P < 0.001, paired t-test) and cyclosporine A-perfused cells and non-significant for the BAPTA-perfused cells.

These results suggested that significant proportion of neocortical excitatory synapses lacks functional P2X receptors in the baseline conditions but activation of glial cells can trigger a recruitment of P2XR into synapses. This process could involve the release of ATP and activity- and Ca^2+^ dependent trafficking of P2XRs, similar to activity-dependent enhancement of the single-bouton purinergic currents (Fig. S2). To verify this, we looked at the effect of NA on purinergic EPSCs in longer time scale which allowed to explore their quantal behavior (Fig. 7). We found out that activation of astroglial α1-adrenoreceptors caused a long-lasting (but still transient) increase in the amplitude of single-axon EPSCs recorded at −80 mV in presence of DNQX (Fig. 7a). This enhancement of purinergic EPSCs originated from appearance of new quantal currents in the type I neurones and increase in the quantal size of purinergic currents in the type II cells (Fig. 7b,c). The facilitation of purinergic EPSCs was significantly inhibited by intracellular perfusion of neurones with BAPTA (Fig. 7a,d), similarly to experiments with single boutons (Fig. S2). Importantly, facilitation was not affected by the intracellular perfusion with PP2B inhibitor (Fig. 7d). Taken together with lack of significant changes in the amplitude of basal mEPSCs (Fig. 5e), this result argues against the role for PP2B-PSD95 cascade in the regulation of trafficking of P2X or NMDARs in the neocortical synapses.

These data give a new insight on role for ATP as a gliotransmitter. Astrocyte-derived ATP can modulate NMDA receptor-mediated synaptic signaling via two mechanisms: facilitating the recruitment of P2XRs into synapses thereby amplify the purinergic signaling and directly down-regulating synaptic NMDARs very likely, through perisynaptic P2XRs. Our data show that glia-driven purinergic modulation of NMDARs relies on PP2B-PSD-95 cascade (Figs 5 and 6) and strongly support the importance of this pathway for activity-dependent modulation of strengths of neocortical synapses.

### Implication of purinergic modulation of NMDA receptors for synaptic plasticity

**Figure 8:**
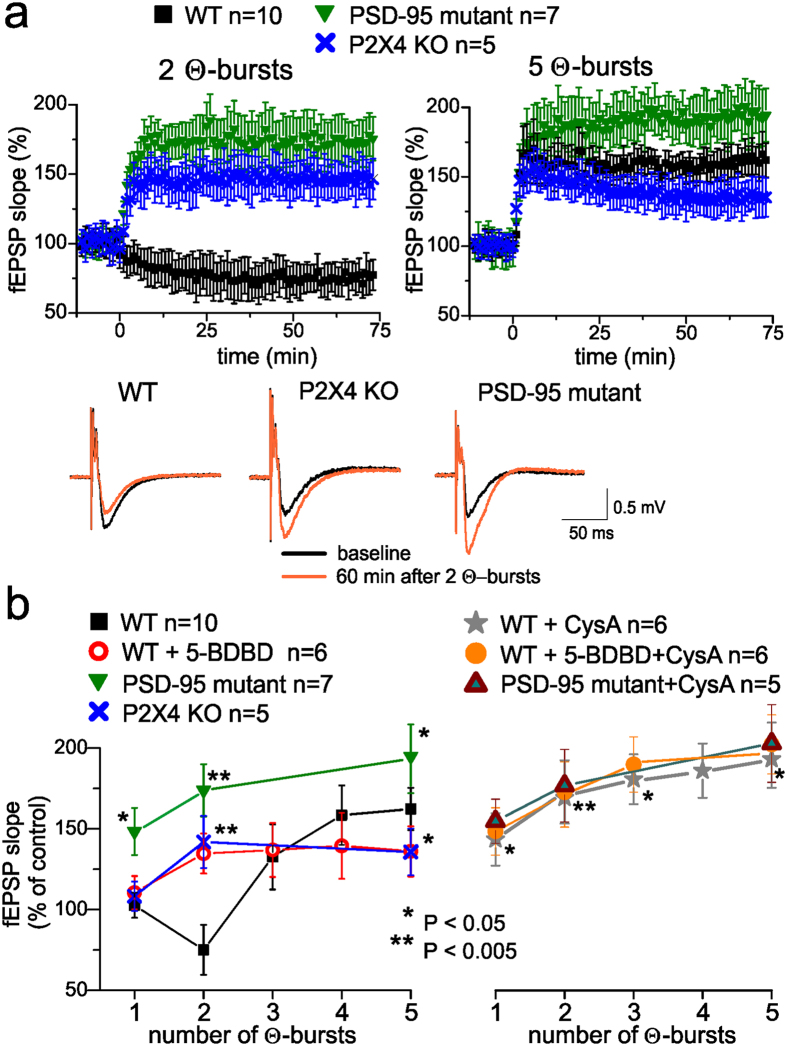
PSD-95 mediated-interaction between P2X and NMDA receptors modulates the long-term synaptic plasticity in the neocortex. (**a**) Long-term potentiation of field EPSPs in the layer 2/3 of somatosensory cortex of wild-type, P2X4 KO and PSD-95 mutant mice was induced by several episodes of theta-burst stimulation (TBS) delivered at zero time. Dots in the graphs represent the average of 6 consecutive EPSPs; data are shown as mean ± SD for number of experiments indicated. Insets show examples of EPSP (average of 20 responses) before and 60 min after TBS. In the wild-type mice, 2 episodes of TBS were unable to induce LTP in the PSD-95 mutant and P2X4 receptor knock-out mice, 2 TBS episodes induced robust LTP. Stronger stimulation (5 TBS episodes) was able to induce LTP in the wild-type, P2X_4_R KO and PSD-95 mutant mice. (**b**) Pooled data on the dependence of the magnitude of LTP in the neocortical layer II/III on the number of TBS episodes under various conditions. Magnitude of LTP was evaluated as relative increase in the fEPSP slope at 60^th^ min, averaged across 10 min time window. Each data point shows mean ± SD for number of experiments indicated. Asterisks (*, **) Indicate the statistical significance of difference with control conditions in the wild-type mice (unpaired t-test). In the right panel, significance is given for the effect of cyclosporine A in wild-type mice; there was no significant difference in the effect of cyclosporine A in the PSD-95 mutants. There is a clear threshold for LTP induction in the wild-type mice, only 3 episodes induced LTP reliably. Knocking-out of significant fraction of P2 purinoreceptors in P2X_4_R KO mice shifted threshold towards weaker stimulus but decreased the net magnitude of LTP. Impairing of control of NMDA receptors in the PSD-95 mutants dramatically decreased the threshold of LTP induction and increased the net magnitude of LTP. Inhibition of PP2B had similar effect as PSD-95 mutation or impairment of P2XR-mediated signalling.

The long-term potentiation (LTP) in the CA1 neurones of the hippocampus was increased in PSD-95 knockout mice^28^,^34^,^35^. It is conceivable therefore, that purinergic modulation of NMDA receptors may also modulate synaptic plasticity in the neocortical neurones. We studied the LTP (by monitoring field EPSPs) in the layer 2/3 of somatosensory cortex of WT, P2X_4_ R KO and PSD-95 KO mice; LTP was induced by several episodes of theta-burst stimulation (TBS). In the WT mice, 2 episodes of did not induce LTP (n = 10) whereas in the P2X4R KO mice LTP was induced in all 5 experiments (Fig. 8a). Similarly, in PSD-95 KO mice, 2 TBS episodes induced robust LTP (n = 7, Fig. 8a). Stronger stimulation (5 TBS episodes) induced LTP in the WT, P2X_4_R KO and PSD-95 KO mice (Fig. 8b), in 10, 5 and 7 experiments respectively. Pharmacological inhibition of P2X4 receptors by selective antagonist 5-BDBD had similar effect as genetic deletion (Fig. 8b).

Our data revealed threshold-like behaviour of LTP induction in the neocortex of wild-type mice (Fig. 8b): 1 episode of TBS did not cause marked changes in the EPSP and 2 TBS episodes even caused moderate depression; only 3 TBS episodes reliably induced LTP. Genetic deletion of significant fraction of P2X receptors in P2X_4_R KO mice shifted threshold towards weaker stimulus but decreased the net magnitude of LTP. Impairment of down-regulation of the NMDA receptor signalling in the PSD-95 mutants dramatically decreased the threshold of LTP induction and increased the net magnitude of LTP. These results are in line with our previous observation of facilitation of LTP induction in CA1 neurones after inhibition of P2X receptors^36^ and previously reported increase in the extent of LTP in the PSD-95 mutant mice^28^. Since our experiments with modulation of NMDAR-mediated synaptic currents suggested the important role for phosphatase 2B, we also tested the impact of cyclosporine A. Extracellular application of cyclosporine A (500 nM) caused significant enhancement of the neocortical LTP in the wild-type mice in all 6 experiments and was occluded in the PSD-95 mutant mice (Fig. 8b). The effect of cyclosporine A also occluded the action of P2XR antagonist (Fig. 8b). These data strongly support the involvement of PP2B-PSD95-NMDAR cascade in the purinergic modulation of neocortical LTP.

Combined, our data show that postsynaptic P2X receptors contribute to the signalling in the population of excitatory neocortical synapses and can moderate activity of NMDA receptors via Ca^2+^-dependent mechanism that involves PSD-95 multi-protein complexes. Purinergic down-regulation of NMDA receptors activity is essential to prevent unnecessary LTP caused by weak or moderate stimuli.

## Discussion

Traditionally, the leading role in purinergic regulation of synaptic transmission in the CNS is assigned to adenosine receptors, which control presynaptic inhibition of neurotransmitter release^1^,^2^. Data presented in this paper highlight an importance of ATP/P2XR-mediated signalling for the modulation of synaptic transmission at postsynaptic site and provide new insights into underlying molecular mechanisms. We demonstrated for the first time that interaction between ATP and glutamate receptors can occur at the level of individual synapse. Furthermore, our data reveal an importance of dynamic cooperation between synaptic and glial ATP release for the signalling in cortical synapses.

Recordings of quantal synaptic currents and immunolabeling of living neocortical neurones (Figs 1 and 2) demonstrate the presence of functional P2XRs in the glutamatergic synapses. The postsynaptic localisation of P2X receptors in excitatory synapses is a most parsimonious and feasible explanation of the large body of data presented in this paper and previous publications, in particular: 1) the existence of distinct fraction of non-glutamatergic mEPSCs and evEPSCs, both in individual synapses and neurones in slices (Figs 1, 2, 3, 6 and 7), refs 11,12,17 and 2) the post-synaptic location of P2X receptors in the dendritic spines of excitatory central synapses shown by immunolabelling and electron microscopy^1^,^8^.

Manifestation of purinergic synaptic currents depends on recruitment of P2XRs to synapses, which can be triggered by synaptic or glial release of ATP (Figs 5, 6 and 7, S2). Dynamic fluctuations in postsynaptic densitiy of P2XRs may explain why, despite evidence of widespread expression of P2X receptors in central neurones, the purinergic component of fast EPSCs are rarely observed and often require stronger stimulation to evoke^8^,^11^,^17^. Our data suggest an important role for astrocyte-derived ATP in the activity-dependent trafficking of synaptic P2X receptors. Our previous^3^ and current results (Figs 5 and 7) show that exocytosis of ATP from astrocytes can activate neuronal P2X receptors which in turn can facilitate additional recruitment of P2X receptors into excitatory synapses by Ca^2+^-dependent mechanisms (Fig. 7). These mechanisms may include Ca^2+^-dependent lateral diffusion^21^, HSP-90-dependent trafficking^22^ and Ca^2+^-regulated endocytosis^20^, as suggested by our experiments in the individual synaptic boutons (Fig. S2).

Activation of P2XRs by low micromolar (i.e. physiologically relevant) concentrations of ATP can significantly down-regulate NMDARs (Figs 3, 4 and 5, Figs S3 and S4; see also ref. 3). Conceptually, purinergic effects on various kinds of NMDAR-mediated responses demonstrate similar functional properties, in particular dependence on increased intracellular Ca^2+^ and phosphatase 2B. One should emphasize that all our observations converge on the post-synaptic locus of purinergic modulation of NMDARs. Our data demonstrate the changes in the quantal size of NMDAR-mediated mEPSCS and evEPSCs (Figs 1, 2, 3 and 6) and do not show any notable changes in the mEPSC frequency or mean quantal content of evEPSCs. Combined with effects of intracellular perfusion with EGTA, BAPTA and CysA (Figs 3, 4, 5 and 6), this strongly supports the post-synaptic mechanism. Interestingly, the effect of intracellular perfusion of neurones with EGTA was notably smaller than effect of BAPTA which has higher affinity and faster kinetics of Ca^2+^-binding (Figs 4 and 5, S5). This suggests that co-localization of P2X and NMDA receptors within dendritic spines may be important for their efficient Ca^2+^ -dependent interaction.

Recent works highlighted the importance of AKAP5-mediated recruiting of PP2B to PS5–95 complex for regulation of AMPAR trafficking and showed the involvement of this pathway in the hippocampal LTD^27^,^31^,^32^. Putatively, P2X receptors also might affect synaptic plasticity by engaging this cascade. However, the physiological impact of purinergic modulation of AMPA receptors can be complex and depend on physiological and regional context. For instance, PKA, which is also bound by AKAP5, can counterbalance the action of PP2B on GluR1 receptors^27^. In general, both by AKAP5/PSD95 mechanism and Ca^2+^-entry through P2X receptors can exert both positive and negative effects on the activity and/or trafficking of AMPA receptors^15^,^16^,^37^. In particular, our data (Fig. S5) suggest activation of P2X receptors in the neocortical neurones can cause moderate increase in the AMPAR receptor-mediated EPSCs in the Ca^2+^ dependent- but PP2B-independent manner. So, one could expect that abolishing of this positive modulation of AMPARs, e.g. in PSD-95 mutant or P2X4 KO mice would lead to deficit rather than enhancement in the LTP (Fig. 8). A further investigation of P2X-mediated modulation of AMPARs is surely worthwhile but it goes beyond the topic of the present paper.

Our observations comply with reports of Ca^2+^-dependent inactivation of NMDARs in hippocampal neurones^23^,^24^,^38^. Both phosphatase- and Ca^2+^-dependent inactivation were implicated in the reduction of fractional Ca^2+^-permeability of GluN2B subunit-containing NMDARs^24^, highly relevant for regulation of synaptic plasticity. In the hippocampal CA1 pyramidal neurones, high density of GluN2B containing receptors was observed only in younger animals^24^. In the neocortex, the GluN2B-contaning receptors contribute up to 40% to the NMDAR component of synaptic transmission even in mature age, as evidenced by significant effect of ifenprodil on the EPSCs^39^. Thus, activity-dependent plasticity of GluN2B subunits may have even greater importance in the neocortex.

Previously^24^,^38^, Ca^2+^-dependent inactivation was suggested to serve merely as a negative feedback loop controlling activity of NMDARs. Our data indicate that this inactivation is an element of wider regulatory mechanism, which can be activated by variety of pathways, including glial release of ATP. Furthermore, we showed that Ca^2+^-dependent modulation of NMDARs relies on interactions with PSD-95 multi-protein complex (Figs 4 and 5).

The ability of postsynaptic P2XRs to engage this powerful mechanism and regulate glutamate receptors can confer an important (and yet to be fully understood) physiological role to the ATP-mediated synaptic transmission in the brain. The PSD-95 plays a critical role in the binding of the NR2 subunits of NMDAR to multi-protein complexe which also involves phosphatase 2B^25^,^26^. Activity-dependent plasticity of hippocampal synapses is up-regulated in PSD-95 KO mice^28^,^35^ leading to severe impairment of different forms of learning^28^,^34^,^40^. Possibly, deficient de-phosphorylation of NMDARs could make a large contribution to the excessive up-regulation of synaptic plasticity. Our data (Fig. 8) support this hypothesis. Coupling of NMDA receptors to PSD-95 and multi-protein complex may be essential for bi-directional modulation of synaptic strength which, in turn, is essential for proper function of synaptic networks underlying learning and memory^28^.

This pathway of down-regulation of NMDARs can be exploited by postsynaptic P2XRs which can provide a Ca^2+^-influx and activate PP2B. The P2XRs-mediated “moderation” of excitatory synapses can help avoiding “unwanted” or “excessive” LTP, i.e. reduce number of errors. The P2XR-mediated endocytosis of AMPA receptors^16^ could also contribute to this mechanism. By setting up the lower level of activity of an individual synapse and/or by decreasing the number of potentiated synapses, the above mechanisms can also increase a dynamic range of potentiation thus being beneficial for optimal memory storage. These cascades may be triggered by ATP released either from synaptic terminals (Figs 3 and 4) or from astrocytes (Figs 5 and 6)^3^,^16^ in response to a local network activity. Purinergic down-regulation of NMDARs exhibits an integrative behaviour: it requires a certain period of P2X receptors activity (Figs 3, 4 and 5) but relaxes rather slowly afterwards. Retention of inactivation of NMDARs arises, very likely, from the Ca^2+^-regulated de-phosphorylation. Astrocyte-induced Ca^2+^-dependent recruitment of P2XRs (Fig. 7 and Fig. S2) is another mechanism of modulating the strength of individual synapse (Fig. 6). Thus, synergism between synaptic and glial release of ATP (Fig. 6) is instrumental for integrative behaviour of glial modulation of synaptic transmission which in turn contributes to brain metaplasticity^7^.

Our data also highlight a new role for ATP as a gliotransmitter. Astrocyte-derived ATP can modulate NMDAR-mediated synaptic signaling via two mechanisms: facilitating the recruitment of P2XRs into synapses thereby amplifying the purinergic signaling and directly down-regulating synaptic NMDARs very likely, through perisynaptic P2XRs.

To conclude, our results demonstrate that P2XRs are present in the neocortical glutamatergic synapses and can down-regulate NMDARs via Ca^2+^-dependent de-phosphorylation. An engagement of neuronal P2XRs into activity-dependent regulation of glutamatergic receptors can underlie feedback loops controlling both the short-term and homeostatic synaptic plasticity. The relatively slow integrating action of P2XRs, observed in our experiments, agrees with recently suggested hypotheses of slow neuromodulatory role played by P2X receptors in the brain^8^ and slow integrative action of ATP as a gliotransmitter^2^. It becomes evident now that modulatory action of postsynaptic P2X receptors can be very important for the function of central synapses.

## Methods

All animal work has been carried out in accordance with UK legislation (ASPA) and “3R” strategy; all experimental protocols were approved by University of Warwick Ethical Review Committee and Animal Welfare Committee. Experiments were performed on neurones of somato-sensory cortex of 5–8 weeks old C57BL/6 mice and transgenic mice with knockout of P2X4 receptors^30^ (P2X4 KO), PSD-95 KO mutant mice lacking the PDZ-domain of PSD-95 complex^28^, dn-SNARE transgenic mice with impairment of astroglial exocytosis^5^,^29^,^30^. Genotypes of animals were verified by PCR from the ear samples. The genetic background for all transgenic strains used was C57BL/6 mice. Data obtained in the C57BL/6 mice did not differ significantly from data obtained in the WT-littermates of P2X_4_R KO, PSD-95 KO and dn-SNARE mice of the same age (n = 4–5 for each type of experiments). For clarity, all data referred here as wild-type were reported solely for C57BL/6 mice.

### Slice and cell preparation

Mice were anaesthetized by halothane and then decapitated, in accordance with UK legislation. Immediately after decapitation, mouse head were placed into ice-cold physiological saline containing (mM): NaCl 130, KCl 3, CaCl_2_ 0.5, MgCl_2_ 2.5, NaH_2_PO_4_ 1, NaHCO_3_ 25, glucose 15, pH of 7.4 gassed with 95% O_2_ −5% CO_2_; scull was open and brains removed within 30–40 sec. Transverse slices (280 μm) were cut at 4 °C and then placed in physiological saline containing (mM): NaCl 130, KCl 3, CaCl_2_ 2.5, MgCl_2_ 1, NaH_2_PO_4_ 1, NaHCO_3_ 22, glucose 15, pH of 7.4 and kept at 34C for 40 min, after then slices were kept at room temperature for 1–4 h prior the cell isolation and recording.

Neocortical pyramidal neurones were acutely isolated using the modified “vibrating ball” technique^4^,^6^,^18^. The glass ball (200 μm diameter) was moved slowly some 10–50 μm above the slice surface, while vibrating at 100 Hz (lateral displacements 20–30 μm). This technique preserves the function of membrane proteins and therefore is devoid of many artefacts of enzymatic cell isolation and culturing procedures. Non-enzymatic procedure of cell isolation also preserves significant number of functional synapses on the somatic and dendritic membranes^6^,^18^. The composition of external solution for all isolated cell experiments was (mM): 135 NaCl; 2.7 KCl; 2.5 CaCl_2_; 1 MgCl_2_; 10 HEPES, 1 NaH_2_PO_4_, 15 glucose, pH adjusted with NaOH to 7.3.

### Fluorescent microscopy

For labelling of synaptic boutons on acutely isolated neurones with FM1–43 dye, fluorescent imaging was performed with aid of IX51 inverted microscope and epifluorescent illumination via UPLSAPO 60XW/NA1.2 objective (Olympus, Tokyo, Japan). Cells were constantly illuminated at 480 ± 10 nm using OptoLED light source (Cairn Research, Faversham, UK), fluorescence was measured at 535 ± 25 nm. The fluorescent images were recorded using Retiga 2000R enhanced CCD camera (*QImaging*, Canada); exposure time was 35 ms at 2X2 binning.

In some experiments (Fig. 1, Fig. S5), two-photon imaging of neurones was performed using Zeiss LSM-7MP multi-photon microscope coupled to the SpectraPhysics MaiTai pulsing laser; experiments were controlled by ZEN LSM software (Carl Zeiss, Germany). Images were further analyzed off-line using ZEN LSM (Carl Zeiss) and ImageJ (NIH) software. For immunolabelling of postsynaptic densities and P2X and NMDA receptors, living acutely isolated neurones were incubated with 0.2 μg/ml of following antibodies: mouse monoclonal anti-PSD95 (6G6-1C9, Abcam), rabbit polyclonal anti-P2X1, rabbit polyclonal anti-P2X4 and rabbit polyclonal anti-NR1 (Alomone Labs). Prior to cell loading, antibodies were conjugated to the green fluorescent dye Atto488 (P2X1, PSD-95) or red fluorescent dye Atto594 (P2X4, NR1) using Lighting-Link antibody conjugation system (Innova Bioscience, Cambridge, UK) accordingly to the manufacturer’s protocol. Antibodies to P2X_1_R, P2X_4_R and NR1 subunits, which recognize extracellular epitopes, were applied to living neurones directly; antibodies to PSD-95 were conjugated with BioPORTER protein delivery reagent (Genlantis, San Diego, CA) 10 min prior to incubation. Immediately after isolation from the neocortical slice, living neurones were pre-incubated with 2% of normal bovine serum (Sigma) in the extracellular saline for 20 min to block unspecific binding sites. After them, cells were gently washed two times with clean extracellular saline for 5 min and then incubated with antibodies for 40 min at room temperature. After incubation, cells were washed with laminar flow of extracellular solution in the microscope recording chamber for 15 min prior to image recording. Fluorescence was excited at 820 nm; ATTO-488 signal was observed at 520 ± 10 nm, ATTO-594 signal was observed at 590 ± 20 nm. Co-localisation analysis of images was carried out using ImageJ software as described previously^3^,^41^. Briefly, the correlation between green and red fluorescence was evaluated with the aid of intensity correlation quotient (ICQ) and Pearson’s correlation coefficient (Rr) for pairs of different markers. ICQ and Rr were calculated on a basis of product of the relative differences from the mean (PDM) for each pixel using the intensity correlation analysis routine implemented in the ImageJ (MBF plugin package) and described in ref. 41. ICQ was calculated as a relative number of pixels with positive PDM values; Pearson’s coefficient was calculated according conventional definition. The theoretical limits for ICQ and Rr are ±0.5 and ±1 correspondingly; random staining should show ICQ and Rr around 0, positive values are characteristic for dependent staining.

### Electrophysiological recordings

Whole-cell voltage clamp recordings from neocortical neurones were made with patch pipettes (4–5 MΩ for) filled with intracellular solution (in mM): 110 CsCl, 10 NaCl, 10 HEPES, 5 MgATP, 0.1 EGTA, pH 7.35. In some experiments 0.1 mM EGTA was substituted with 3 mM BAPTA or 10 mM EGTA and 1 mM CaCl_2_ to clamp Ca^2+^ concentrations at physiological resting levels (about 80 nM). Currents were monitored using an AxoPatch200B patch-clamp amplifier (Axon Instruments, USA) filtered at 2 kHz and digitized at 4 kHz. Experiments were controlled by PCI-6229 data acquisition board (NI, USA) and WinFluor software (Strathclyde University, UK); data were analyzed by self-designed software. Liquid junction potentials were compensated with the patch-clamp amplifier. Series and input resistances were respectively 5–7 MΩ and 500–1100 MΩ; both series and input resistance varied by less than 20% in the cells accepted for analysis. For activation of synaptic inputs, axons originating from layer IV-VI neurones were stimulated with a bipolar coaxial electrode (WPI, USA) placed in the layer V close to the layer IV border, approximately opposite the site of recording; stimulus duration was 300 μs. If not stated otherwise, the stimulus magnitude was set 3–4 times higher than minimal stimulus adjusted to activate the single-axon response in the layer II pyramidal neurones as previously described^17^. In some experiments (Figs 7 and 8) the stimulus intensity was set at minimal level, just 10–15% higher in intensity than threshold at which EPSC appeared (typically 0.8–1.2 μA). Minimal level stimulation was set to meet the criteria of single-axon stimulation including all-or-none synaptic response, little variation in EPSC latencies, and absence of change in the mean size or shape of the EPSC with 20–50% increase in stimulus intensity^17^. For induction of long-term plasticity 1 to 5 episodes of theta-burst stimulation (TBS) were used; each TBS episode consisted of 5 pulses of 100 Hz stimulation, repeated 10 times with 200 ms interval (total 50 pulses per episode).

### Stimulation of individual synaptic boutons

After establishing the whole-cell configuration between recording pipette and postsynaptic neurone, individual synaptic boutons were identified with the aid of fluorescence and gradient contrast imaging. To label nerve terminals, acutely isolated neurones were pre-incubated with 2.5 μM FM1–43 for 15 min and then was washed for 15 min. Visual investigation of neurones and synaptic boutons were performed at total magnification of ×600 using green light (535 ± 25 nm) and high-aperture water immersion objective (Olympus, UPLSAPO 60XW/NA1.2). Optical resolution of this system is about 270 nm, which was usually enough to identify synaptic boutons located on the dendrites. The boutons showing FM1–43 fluorescence were chosen for further investigation.

Glass stimulation pipette of inner diameter about 1 μm filled extracellular solution (resistance 8–10 MΩ) was placed close to the identified synaptic bouton, approximately at a distance of 0.5 μm. Movement of the stimulation pipette was controlled by high-resolution and ultra-low drift motorized micromanipulator (PiezoPatch PPM5000, WPI). Stimulating pulses of negative voltage (5–9 V amplitude and 1–5 ms duration) were applied to the pipette via isolated constant-voltage stimulation unit (Digitimer DS2A); stimulation frequency was 0.33 Hz if not directly specified otherwise.

This method of stimulation was successfully used in the past to record single-synapse responses in the cultured hippocampal neurones^19^,^39^. Application of voltage pulses up to 15 V via glass micropipette alters the extracellular potential in a very small area (effective radius about 1 μm) that includes presynaptic area and an underlying patch of postsynaptic membrane. Typically, the magnitude of changes in the extracellular field potential in the immediate vicinity of the presynaptic bouton can reach 50–80 mV^42^ for limited period, defined by the voltage pulse. It has to be noted that effect of stimulation on the postsynaptic membrane of recorded neurones is greatly diminished due to the voltage-clamping, especially at short electronic distances (which is the case in our experiments). Thus, the main effect of the field stimulation is depolarisation of a small (about 1 μm^2^) area of presynaptic membrane which can activate an influx of Ca^2+^ via voltage-gated Ca^2+^ channels^19^ and trigger the fusion of synaptic vesicles. Similar to the previous work^19^,^42^, applying of voltage pulse of 1–5 ms duration to the pipette placed close to the synaptic bouton elicited fluctuating whole-cell postsynaptic currents which disappeared when pipette was shifted for 1–1.5 μm along the dendrite. Disappearance of responses after small displacement of the stimulating pipette off the bouton verified that they were not caused by side-effect of stimulation on postsynaptic membrane. Also, a pre-synaptic origin of responses was verified by changes in the FM1–43 fluorescence of stimulated bouton (see Fig. 4) and dependence of response on the extracellular Ca^2+^ and stimulus duration (Fig. S1).

### Data analysis

All data are presented as mean ± SD, the statistical significance of difference between data groups was tested by two-population t-test, unless indicated otherwise. The spontaneous transmembrane currents recorded in neurones were analysed off-line using methods described previously^3^,^6^,^17^,^43^.

The amplitude and kinetics of synaptic currents were determined by fitting with one or two (where appropriate) theoretical waveforms with monoexponential rise and decay phases. As a rule, mean square error of fit amounted to 5–20% of peak amplitude depending on the background noise.

In analysis of complex evoked EPSCs, containing P2XR and NMDAR-mediated components, two theoretical waveforms were used. Decay time constant of the “fast” component varied in the range of 9 ± 2 ms, decay time of “slow” component varied in the range of 28 ± 8 ms. Rise time constants of both components varied in the range of 1.5 ± 1.0 ms. The amplitudes of components were calculated directly using mean square root routine to provide a best fit for experimental EPSC, as described previously in refs 17,43. This approach was demonstrated to provide a high noise-tolerance and good accuracy in resolving amplitudes of fast and slow components of EPSCs^43^. The amplitude distributions of spontaneous and evoked currents were analyzed with the aid of probability density functions and likelihood maximisation techniques; all histograms shown were calculated as probability density functions. The amplitude distributions were fitted with either multi-quantal binomial model or bi-modal function consisting of two Gaussians with variable peak location, width and amplitude. The decay time distributions were fitted with bi-modal functions. Parameters of models were fit using likelihood maximisation routine. To monitor and analyse the time course of changes in the amplitude and frequency of spontaneous currents, the amplitude and frequency were averaged over the 1 min time window.

## Additional Information

**How to cite this article**: Lalo, U. *et al.* ATP from synaptic terminals and astrocytes regulates NMDA receptors and synaptic plasticity through PSD-95 multi-protein complex. *Sci. Rep.* **6**, 33609; doi: 10.1038/srep33609 (2016).

## Supplementary Material

Supplementary Information
