# Supplementary material for: ATP from synaptic terminals and astrocytes regulates NMDA receptors and synaptic plasticity through PSD-95 multi-protein complex
Source: Sci Rep. 2016 Sep 19;6:33609. doi: 10.1038/srep33609 (PMC5027525; doi:10.1038/srep33609)
Supplement: Supplementary Information [file srep33609-s1.pdf]

ATP from synaptic terminals and astrocytes regulates NMDA receptors and synaptic plasticity through PSD-95 multi-protein complex

U.Lalo, O.Palygin, A.Verkhratsky, S.G.N. Grant and Y. Pankratov

Supporting Information

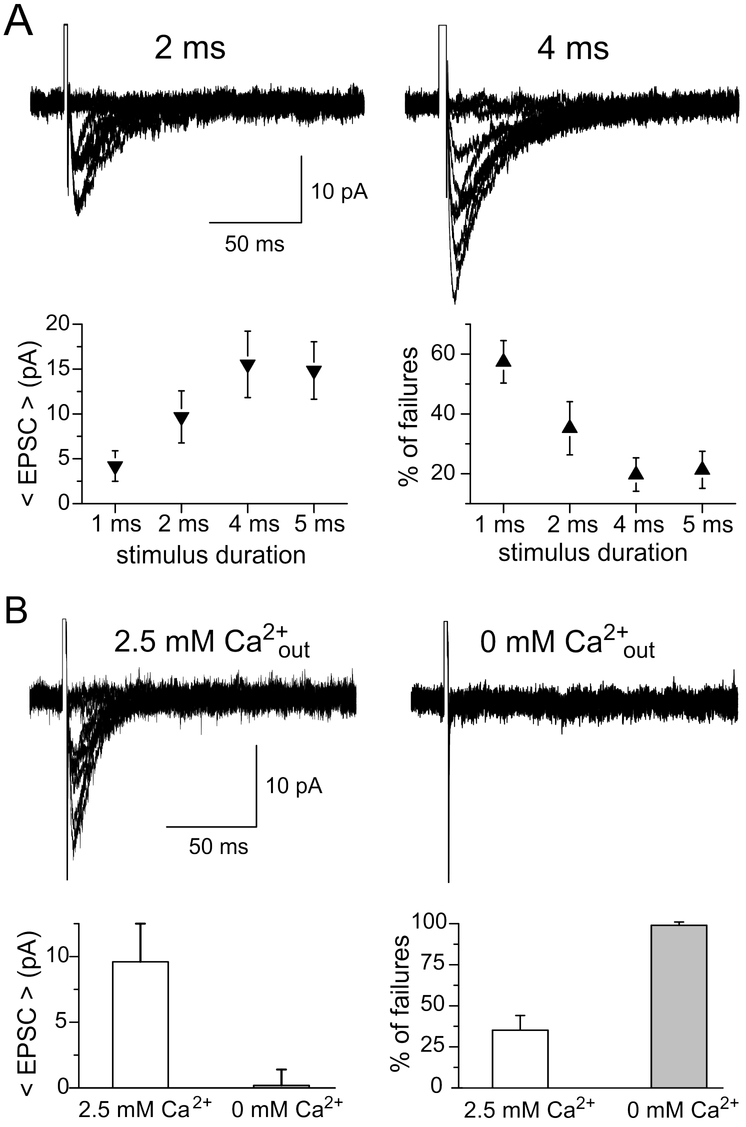

Figure S1

Stimulus and Ca^{2+} - dependence of responses in individual synapses.

Single-bouton evoked synaptic currents (evEPSCs) were elicited by electric field stimulation of identified synaptic bouton as shown in the Figure 2 of main text.

(A) The evEPSCs were recorded at membrane potential of -80 mV in presence of 100 μM picrotoxin at different stimulation duration. Each upper graph shows 10 consecutive individual responses elicited in the same synapse by 2 ms- and 4-ms long stimulation pulse. Lower graphs show the dependence of average evEPSC and proportion failures (responses of zero amplitude) on stimulus duration. Data are shown as mean±SD for 7 glutamatergic synapses.

(B) The evEPSCs were recorded at membrane potential of -80 mV in presence of 100 μM picrotoxin at different concentrations of extracellular Ca^{2+}. Each upper graph shows 10 consecutive responses elicited in the same synapse at normal and zero extracellular Ca^{2+}. Diagrams show the data (mean±SD) on average evEPSC and proportion failures pooled for 5 neurons. Note that no response was elicited at the absence of extracellular Ca^{2+} (100% failures).

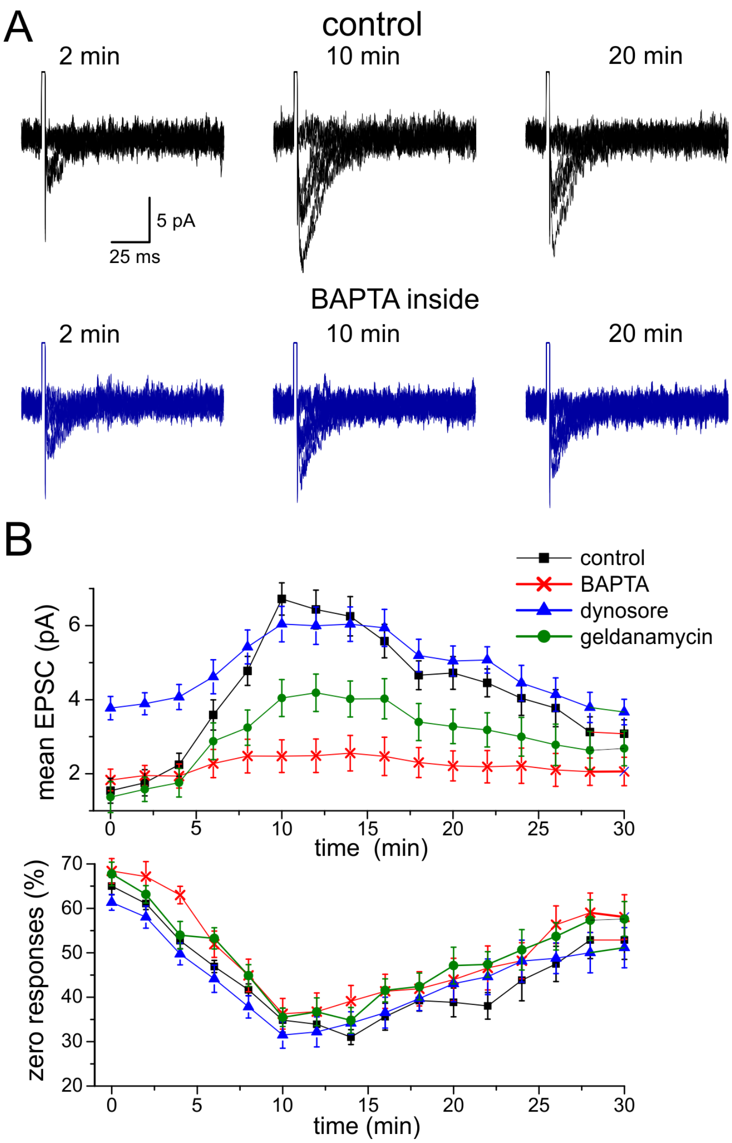

Figure S2

Trafficking and mobility of P2X receptors affect the time-dependence of responses of individual synaptic boutons.

Single-bouton evoked synaptic currents (evEPSCs) were elicited by electric field stimulation of identified synaptic bouton in presence of 100 μM picrotoxin, 50 μM DNQX and 30 μM D-APV. Currents were recorded at membrane potential of -80 mV. Currents recorded under these conditions are mediated by P2X receptors, as discussed in the main text. The initial amplitude of P2X receptor-mediated currents was very small but significantly increased after 10-20 min of continuous stimulation. To dissect the influence of post- and presynaptic mechanisms, we assessed the average probability of zero-responses and applied intracellular agents, which were reported to influence the trafficking and endocytosis of P2X receptors [20-22] via whole-cell recording pipette.

(A) The representative purinergic evEPSCs recorded at different periods of time after establishing whole-cell voltage-clamp recording configuration. Neurons were perfused with intracellular solution containing either just 0.1 mM EGTA (control) or 3 mM BAPTA. Each upper graph shows 10 consecutive individual responses.

(B) The changes in the average amplitude and proportion of failures of synaptic responses recoded in neurons perfused with 0.1 mM EGTA (control), 3 mM BAPTA and 0.1 mM EGTA with addition of inhibitor of heat-shock protein HSP90 geldanamicyin (10 μM) or dynamin inhibitor dynosore (10 μM). Data are shown as mean±SD for 5 experiments.

Intracellular perfusion of neurons with Ca^{2+}-chelating agent BAPTA and inhibitor of heat-shock protein 90 (HSP90) geldanamycin significantly decreased the stimulation-dependent enhancement of purinergic EPSCs. Impairment of dynamin-dependent endocytosis by intracellular dynosore elevated the baseline amplitude of purinergic EPSCs but decreased their further enhancement (Figure S2B). These results go in line with previous observations [20] and suggest that density of P2X receptors at synaptic sites can be strongly influenced by postsynaptic mechanisms such as Ca^{2+}-dependent lateral diffusion [21], HSP-90-dependent trafficking [22] and Ca^{2+}-regulated endocytosis [20]. An increase in the amplitude of EPSCs was accompanied by increase in probability of non-zero synaptic response, which could arise, especially in the initial period, from the elevation of the currents over the threshold of detection. Still, further increase could be also attributed to the enhancement of ATP release during prolonged stimulation due stimulation-dependent recruitment of ATP-containing vesicles. Such enhancement has been reported previously [43]. It is worth noting that after 15-20 min of stimulation, both EPSC amplitude and proportion of non-zero responses declined, most likely due to depletion of ATP-containing vesicles and desensitization of receptors [44].

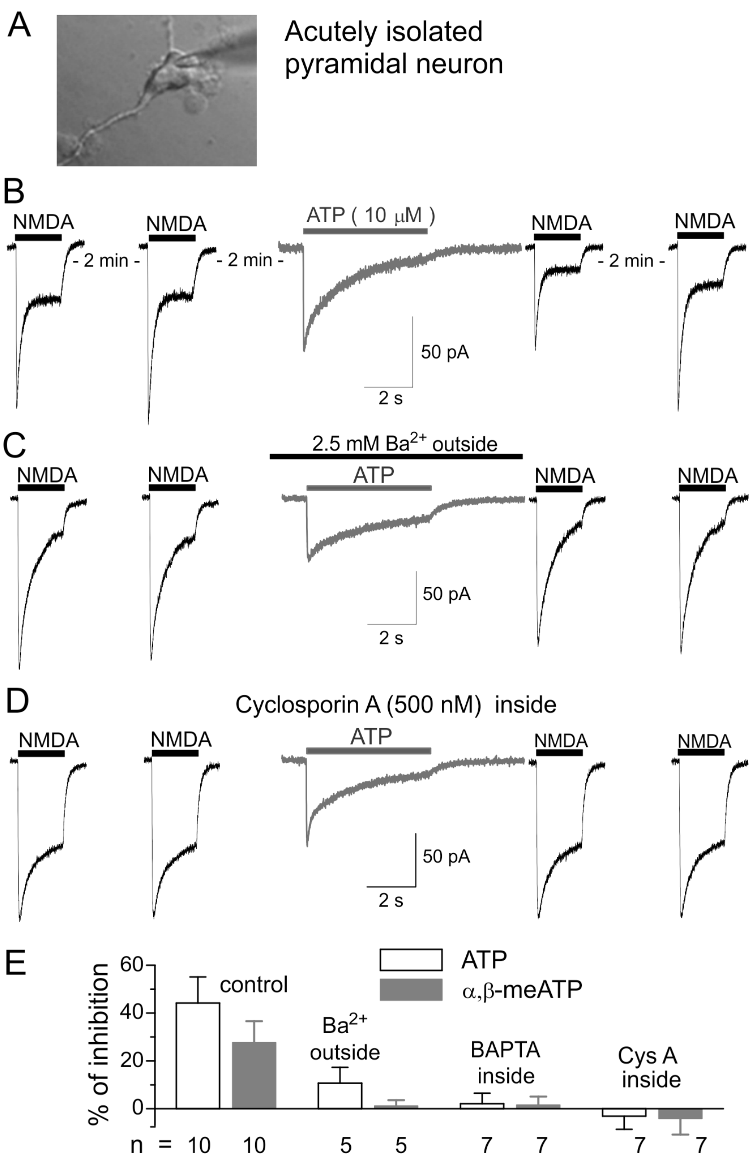

Figure S3

Down-regulation of NMDA receptor-mediated currents in isolated neocortical neurons is Ca^{2+}-dependent.

To explore cross-talk between NMDA and P2X receptors, we elicited the whole-cell currents in acutely isolated neocortical pyramidal neurons (A) by fast application of NMDA (20 μM) and P2XR agonists ATP (10 μM) and α,β meATP (10 μM). Marked responses to both agonists were observed in 58 of 74 cells tested. When NMDA receptor-mediated currents were evoked with a 2 min interval, they had stable amplitudes (panels B-D, first 2 traces). In contrast, the amplitude of NMDA-mediated currents evoked immediately after activation of P2XRs was substantially reduced (B). Inhibition of NMDA receptors reached 42±18% (n=10) with ATP application and 26±13% (n=10) with α,β meATP. Inhibition of NMDA-evoked current was reversible in all cells tested (B). When ATP was applied in the absence of extracellular Ca^{2+} , it inhibited the NMDA currents by only 9.4±7% (C,E); application of α,β-meATP in similar conditions had no effect at all (n=5 cells for both cases). It has to be noted here, that ATP could activate both influx of Ca^{2+} via P2X receptor channels and P2Y receptor-mediated release from intracellular stores whereas α,β-meATP would activate just several subtypes of P2X receptors. Importantly, down-regulation of NMDA receptors was dramatically reduced (D,E) by intracellular perfusion of cells with inhibitor of phosphatase 2B cyclosporine A (500 nM) or Ca^{2+} chelator BAPTA (3 mM). These results are in a good agreement with previous data on Ca^{2+}- and dephosphorylation-dependent inactivation of NMDA receptors in hippocampal neurons [23,34].

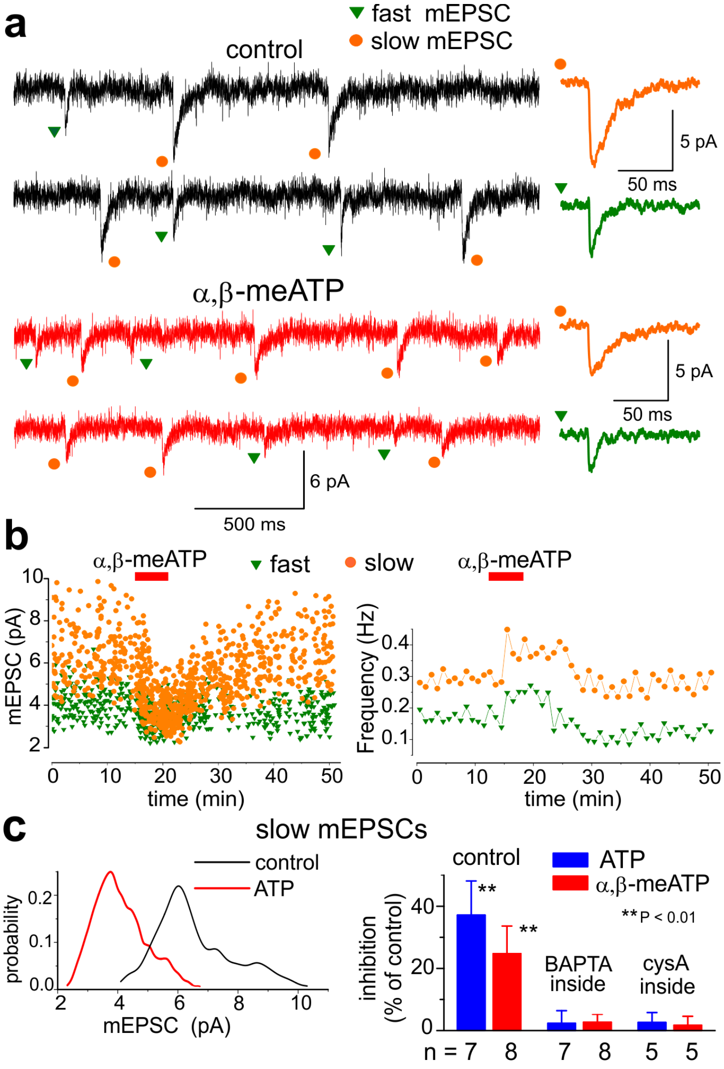

Figure S4 - Activation of P2X receptors causes an inactivation of NMDA-mediated EPSCs in individual neocortical synapses. We mimicked the impact of glial release of ATP on NMDAR-mediated mEPCSs by application of exogenous ATP (10 μM) and its non-hydrolysable analogue α,β-methylene ATP (10 μM). The synaptic currents mediated by P2XRs and NMDARs were separated by their kinetics, correspondingly faster and slower, as demonstrated above (Fig. 1A).

(A) Representative spontaneous synaptic currents recorded in the acutely isolated neocortical pyramidal neurone before (control) and after application of α,β-meATP . Recordings were performed at membrane potential of -40 mV in the presence of 1 μM TTX, 100 μM picrotoxin and 50 μM NBQX. Dots indicate the faster (green) and slower (orange) mEPSCs, mediated correspondingly by P2X and NMDA receptors, as demonstrated in the Figure 2.

(B) The time course of amplitude and frequency of fast (green) and slow (orange) mEPSCs. Each dot in the amplitude graph (left panel) shows individual mEPSCs, each dot in the right panel shows an average frequency of mEPSCs recorded within 1 min time window. Application of ATP caused considerable decrease in the amplitude but not in the frequency of slow glutamatergic mEPSCs.

(C) Amplitude distributions of the slow mEPSCs recorded in the control and during first 8 min after application of ATP. Note the significant left-ward shift in the distribution which indicate the decrease in the quantal amplitude of slow synaptic currents supporting the postsynaptic mechanism of effect.

(D) Pooled data on the relative reduction of the amplitude of NMDA receptor-mediated slow mEPSCs by ATP and α,β-methylene ATP applied in the control and in presence of intracellular BAPTA (3 mM) and cyclosporine (500 nM). Data are shown as mean ± SD for the number of experiments indicated. The reduction of the NMDAR-mediated currents was statistically significant with $P < 0.01$ (one-population t-test) for both purinergic agonists. Note the dependence of the inactivation of NMDA currents on intracellular Ca^{2+} and significant contribution of P2X receptors, indicated by the effect of α,β-meATP.

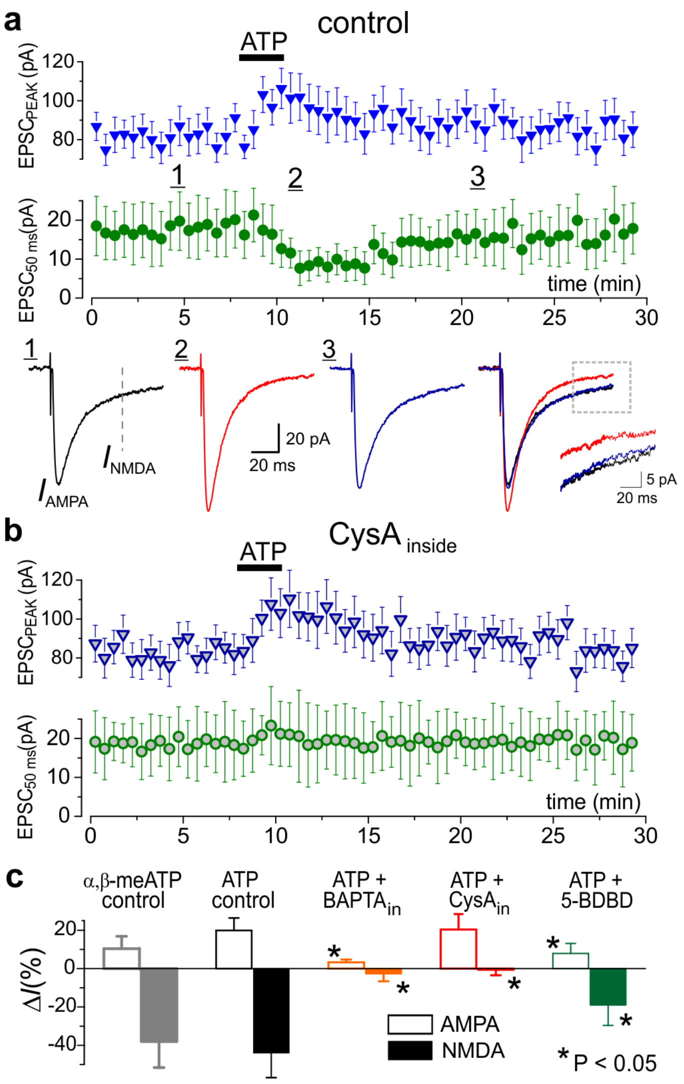

Figure S5 Activation of P2X receptors causes an inactivation of NMDA receptors-mediated component of EPSCs in neocortical slices. P2X receptors were activated by 3 min-long bath application of exogenous ATP (10 μM) and its non-hydrolysable analogue α,β-methylene ATP (10 μM) in the presence of adenosine A1 receptor inhibitor.

(a,b) Excitatory synaptic currents were recorded in the pyramidal neurones of cortical layer II/III in the presence of 100 μM picrotoxin and 10 μM DCPPX at membrane potential of -40 mV; EPSCs were stimulated at 0.2 Hz at -40 mV. The AMPAR - and NMDAR-mediated components of synaptic response were evaluated correspondingly at the peak (I_{AMPA}) and at 50th msec after the peak (I_{NMDA}) as described previously [31,42] and illustrated in lower graph in the panel **(a)**; separation of the component was verified by application of NBQX (20 μM) at the end of experiment.

(a) The time course of changes in the amplitude of AMPAR- and NMDAR-mediated components after application of ATP under control conditions; each dot represents mean ± SD for EPSCs recoded within 30 sec-long period. Lower panel shows the corresponding examples of EPSCs (average of 12 consecutive responses) recorded before and after application of ATP at the moments indicated. Note that decrease in the NMDAR-mediated current was accompanied by an increase in the AMPA-component.

(b) Similar experiment was performed during intracellular perfusion of pyramidal neuron with PP2B inhibitor cyclosporine A (500 nM).

(c) Pooled data on the relative changes in the AMPAR and NMDAR-mediated synaptic currents measured 10 min after application of ATP or α,β-methylene ATP to neocortical slices of the wild-type mice. Data are shown as mean ± SD for 6 experiments for each condition. Asterisks (*) indicate statistically significance of the difference from control conditions (unpaired t-test). Smaller effect of the α,β-methylene ATP and inhibition with 5-BDBD (10 μM) supports the involvement of P2X4 receptors. Note the opposing effects of purinoreceptors on AMPA and NMDA receptors. Intracellular BAPTA strongly inhibited purinegic modulation of both AMPAR-mediated and NMDAR-mediated currents whereas cyclosporine A affected only down-regulation of NMDARs. These data suggest that P2X receptors can modulate AMPAR-component of synaptic response by Ca^{2+} dependent mechanism which do not involve PP2B, this result goes in line with previous observations [15,16].

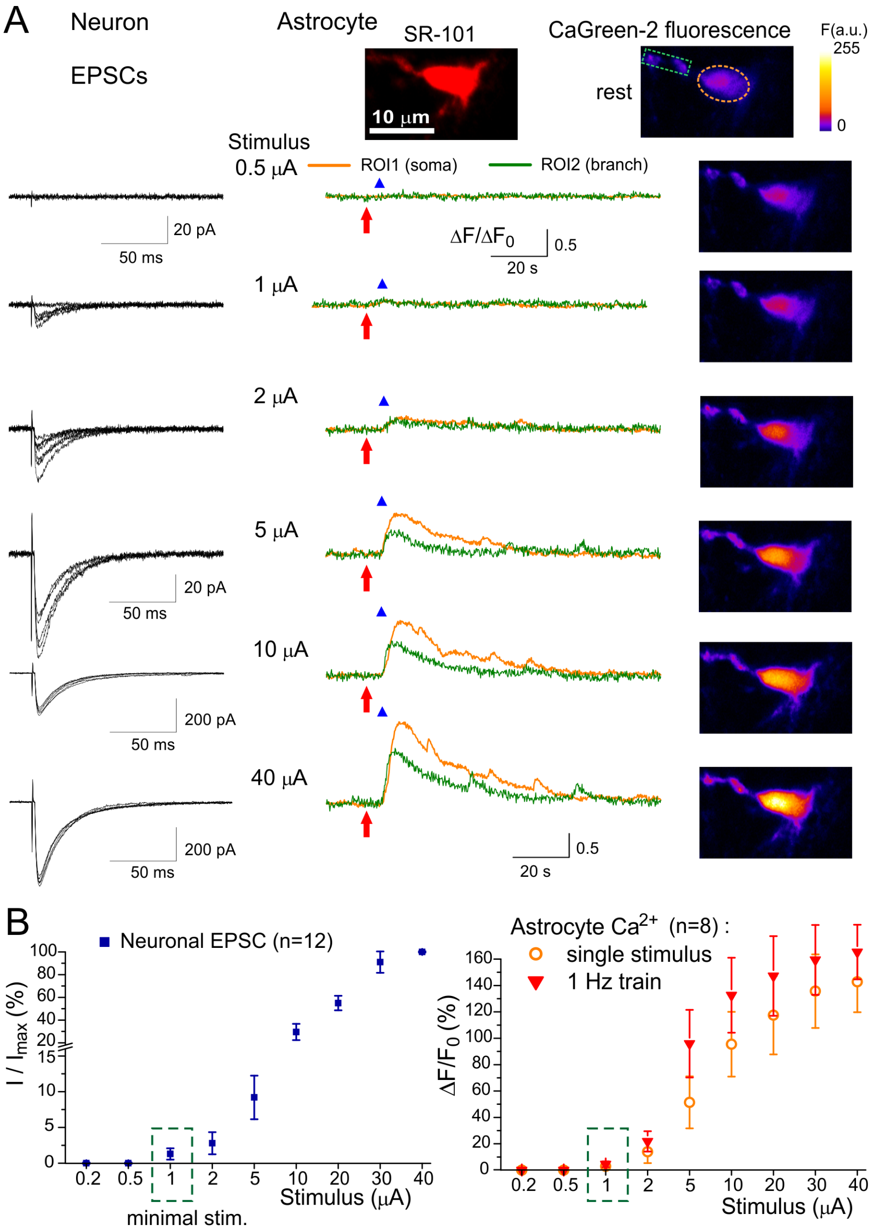

Fig S6

Stimulus-dependence of synaptic currents and astrocyte Ca^{2+}-signalling in the neocortex.

Excitatory synaptic currents (EPSCs) and Ca^{2+}-transients were evoked correspondingly in pyramidal neurons and astrocytes of the layer II/III by field stimulation of cortical afferents in the layer V (see Methods) at different stimulus strength. Astrocytes were loaded with Ca^{2+}-probe Ca-Green 2 AM and identified by the staining with sulpharhodamine 101 and electrophysiological characterization at the end of experiments [45]. The Ca-Green 2 fluorescent imaging in astrocytes was performed using LSM 7 multi-photon microscope at 5 frames/sec. The whole-cell EPSCs in pyramidal neurons were recorded at -80 mV in the presence of 100 μM picrotoxin.

(A) The representative EPSCs, Ca^{2+} -transients and fluorescent images recorded at different stimulus strength as indicated. *Left* column: each panel shows six consecutive individual EPSCs. Note the 10-times larger scale at stimuli of 10 and 40 μA. *Middle* column: time course of Ca-Green 2 fluorescence averaged over the ROIs indicated at the astrocyte image recoded before stimulation (rest), the moments when stimuli were delivered are indicated by red arrows. The scale is the same for all stimuli. *Right* column: the pseudo-color images show the peak Ca-Green 2 fluorescence (average of five frames) recorded at the moments indicated with red triangles.

(B) The pooled data (mean ± SD) on stimulus dependence of EPSCs and astroglial Ca^{2+}-signalling for the number of cells indicated. Astroglial Ca^{2+} signal was evaluated as a peak Ca-Green 2 fluorescence averaged over the whole cell image for single stimulus and train of 5 stimuli delivered at 1 Hz. Dotted lines indicate the region of stimulus strength used for a single-axon stimulation used in the experiments shown in Figures 7 and 8 of main text (see also Methods). Note that stimuli of 1-2 μA reliably evoke quantal EPSCs without evoking the notable Ca^{2+}-elevation in astrocytes. At the same time, stimulus strength of 5 μA (typical for experiments shown in Figs. 5 and 9 of main text) is capable to evoke significant astroglial signal.
